# What are microsatellites and how to choose the best tool: a user-friendly review of SSR and 74 SSR mining tools

**DOI:** 10.3389/fgene.2024.1474611

**Published:** 2024-11-13

**Authors:** Sandy Ingrid Aguiar Alves, Carlos Willian Dias Dantas, Daralyns Borges Macedo, Rommel Thiago Jucá Ramos

**Affiliations:** ^1^ Institute of Biological Sciences, Federal University of Minas Gerais, Belo Horizonte, Minas Gerais, Brazil; ^2^ Laboratory of Simulation and Computational Biology — SIMBIC, High Performance Computing Center — CCAD, Federal University of Pará, Belém, Pará, Brazil; ^3^ Laboratory of Bioinformatics and Genomics of Microorganisms, Institute of Biological Sciences, Federal University of Pará, Belém, Pará, Brazil

**Keywords:** microsatellites, short tandem repeats, simple sequence repeats, SSR tools, molecular markers, computational tools

## Abstract

Microsatellites, also known as SSR or STR, are essential molecular markers in genomic research, playing crucial roles in genetic mapping, population genetics, and evolutionary studies. Their applications range from plant breeding to forensics, highlighting their diverse utility across disciplines. Despite their widespread use, traditional methods for SSR analysis are often laborious and time-consuming, requiring significant resources and expertise. To address these challenges, a variety of computational tools for SSR analysis have been developed, offering faster and more efficient alternatives to traditional methods. However, selecting the most appropriate tool can be daunting due to rapid technological advancements and the sheer number of options available. This study presents a comprehensive review and analysis of 74 SSR tools, aiming to provide researchers with a valuable resource for SSR analysis tool selection. The methodology employed includes thorough literature reviews, detailed tool comparisons, and in-depth analyses of tool functionality. By compiling and analyzing these tools, this study not only advances the field of genomic research but also contributes to the broader scientific community by facilitating informed decision-making in the selection of SSR analysis tools. Researchers seeking to understand SSRs and select the most appropriate tools for their projects will benefit from this comprehensive guide. Overall, this study enhances our understanding of SSR analysis tools, paving the way for more efficient and effective SSR research in various fields of study.

## 1 Introduction

Microsatellites (MS) are highly polymorphic regions of DNA widely employed in fields such as oncology, forensics, plant breeding, comparative genomics, and microorganism research (Chambers and MacAvoy, 2000; Das et al., 2019). However, initiating studies on these repetitive elements can be challenging due to the fragmented nature of the existing knowledge. This fragmentation may arise from several factors, including the use of different synonymous terms, the variety of available methodologies, and the inherent limitations of the technique, all of which can pose challenges for researchers in the field (Vieira et al., 2016).

It has been approximately 35 years since the term Microsatellite (MS) was first introduced (Litt and Luty, 1989). Since then, these repetitive sequences have been referred to by diverse names. The most recurrent synonyms used almost interchangeably to MS in the literature and throughout this paper are Simple Sequence Repeats (SSRs) and Short Tandem Repeats (STRs). However, they are also mentioned in studies regarding Variable Number Tandem Repeats (VNTR), Simple Sequence Tandem Repeats (SSTR), Inter Simple Sequence Repeats (ISSR), Simple Sequence Length Polymorphisms (SSLP), and Sequence-Tagged Microsatellites (STMS) (Mudunuri et al., 2010b; Marwal et al., 2014; Jilani and Ali, 2022; Park et al., 2022).

Traditional methodologies for SSR exploration involve genomic fragmentation, microsatellite enrichment, and clone library construction, utilizing techniques such as PCR amplification in biological samples, gel electrophoresis, and Sanger sequencing. These methods have long been the cornerstone of wet laboratory experiments and are still widely employed in fields such as forensic identification, where length polymorphisms of certain STR markers in alleles are analyzed to compare individuals and determine, for example, paternity (Haddrill, 2021). While these techniques have significantly advanced the field, they often face practical challenges, including the need for laboratory infrastructure, equipment, reagents, and specific primers for SSR analysis. These factors contribute to high costs and labor-intensive procedures, particularly in large-scale studies (Thiel et al., 2003; Oliveira et al., 2008; Churbanov et al., 2012; Metz et al., 2016; Das et al., 2019; Guang et al., 2019; Luo et al., 2020).

The rise of Next-Generation Sequencing (NGS) platforms has been progressively shifting the emphasis from studying a few markers towards whole-genome analysis and population genetics (Vieira et al., 2016; Alves et al., 2022). Genome sequencing has become faster and more affordable, enabling the sequencing of hundreds of genomes and transcriptomes of key organisms (Guang et al., 2019), while generating large amounts of publicly available sequence data in databases (Alves et al., 2023). The direct sequence to SSR approach offers a distinct advantage over enrichment-based strategies, as it eliminates the need for prior selection of specific motifs or prior knowledge of the genomic SSR content (Castoe et al., 2012).

*In silico* analysis, which involves exploring microsatellites directly from sequence data through computational techniques, has emerged as a promising approach to manage the significant volume of data and expedite processing while maintaining precision (Oliveira et al., 2008; Vieira et al., 2016). This method is becoming increasingly prevalent for microsatellite discovery and marker development, as it is more efficient and cost-effective (Sharma et al., 2007; Churbanov et al., 2012; Vieira et al., 2016; Umang et al., 2022). A key advantage is that users can freely download genomes from databases such as NCBI and use free computational tools - referred to here as SSR tools - to perform microsatellite identification (also known as SSR mining or prospection), analysis, and even develop new markers, design primers, and simulate primer amplification *in silico* (da Costa Pinheiro et al., 2022). However, it is important to recognize that despite the potential of *in silico* SSR analysis, wet lab methodologies continue to play a crucial role, particularly in validating computational predictions and providing critical biological insights (Li et al., 2020).

Regarding the identification of microsatellites through computational methods, numerous tools have been developed over the years, primarily to address gaps identified in existing software, allowing for more sensitive and efficient analysis of these repetitive elements (Pickett et al., 2016). Some older but well-established tools continue to be widely cited in the literature, such as TRF (Benson, 1999) and RepeatMasker (Tarailo-Graovac and Chen, 2009), while new ones are continually emerging, including EasySSR (Alves et al., 2023) and MegaSSR (Mokhtar et al., 2023). The diversity of these tools is evident in various aspects such as execution, input type, and outputs, offering researchers a broad range of options tailored to different datasets and experimental needs (Merkel and Gemmell, 2008; Mathur et al., 2020).

The abundance of tools available for SSR analysis presents a challenge for researchers seeking the most suitable option for their specific needs. This situation often initiates a cycle: a researcher searching for a tool may encounter numerous options but feel uncertain about which to choose. Consequently, many tend to select tools based on their visibility in methodologies or high citation rates. While these tools might be perfectly suitable, some researchers may find them lacking, necessitating adaptations to their work or the development of new tools, often unaware that alternatives with the desired functions may already exist. The fragmentation of the SSR literature further complicates this process, making it challenging to identify these alternative tools. This cycle not only boosts the citation counts of popular tools but also leads to the continual emergence of new tools, many of which are innovative and more efficient, while others may offer redundant functions and performance (Mudunuri et al., 2010b; Lim et al., 2013).

Despite the availability of insightful reviews on microsatellite prediction software over the past decades, the accelerating pace of development in the field leads to rapid information obsolescence (Leclercq et al., 2007; Sharma et al., 2007; Merkel and Gemmell, 2008; Mudunuri et al., 2010b; Lim et al., 2013; Zribi et al., 2016; Mathur et al., 2020). For example, new tools have been introduced, and some of the listed tools are no longer operational, emphasizing the need for ongoing reviews to ensure that the information presented remains current and comprehensive regarding the maximum number of available tools (Mathur et al., 2020).

This paper presents an exhaustive examination of the current state of the art microsatellites mining tools. To provide guidance to users of SSR tools, it comprises two main sections: “Section 1: A guide to what are microsatellites” – the SSR section, and “Section 2: A guide to tools for identifying microsatellites” – the tools section. Advanced readers may focus on the section of interest without detriment to understanding if they skip one of the sections. However, for readers seeking a comprehensive understanding, reading the SSR section is recommended to grasp the main concepts that will aid in understanding what SSRs are and assist in interpreting many of the terms used in the parameters and outputs of the SSR tools. In the tools section, the goal was to gather as many SSR tools as possible, group these tools into subgroups to facilitate analysis, and provide informational tables highlighting various criteria that can influence tool selection. Lastly, the discussion highlights key factors influencing tool selection, addressing the question, “How to choose the best tool?”. The purpose of this article is not to indicate the best SSR tools but to serve as a guiding resource for users, helping them understand what microsatellites are and assisting them in the conscientious selection of the most suitable tool for their specific research requirements.

## 2 Section 1: a guide to what are microsatellites

**FIGURE 1:**
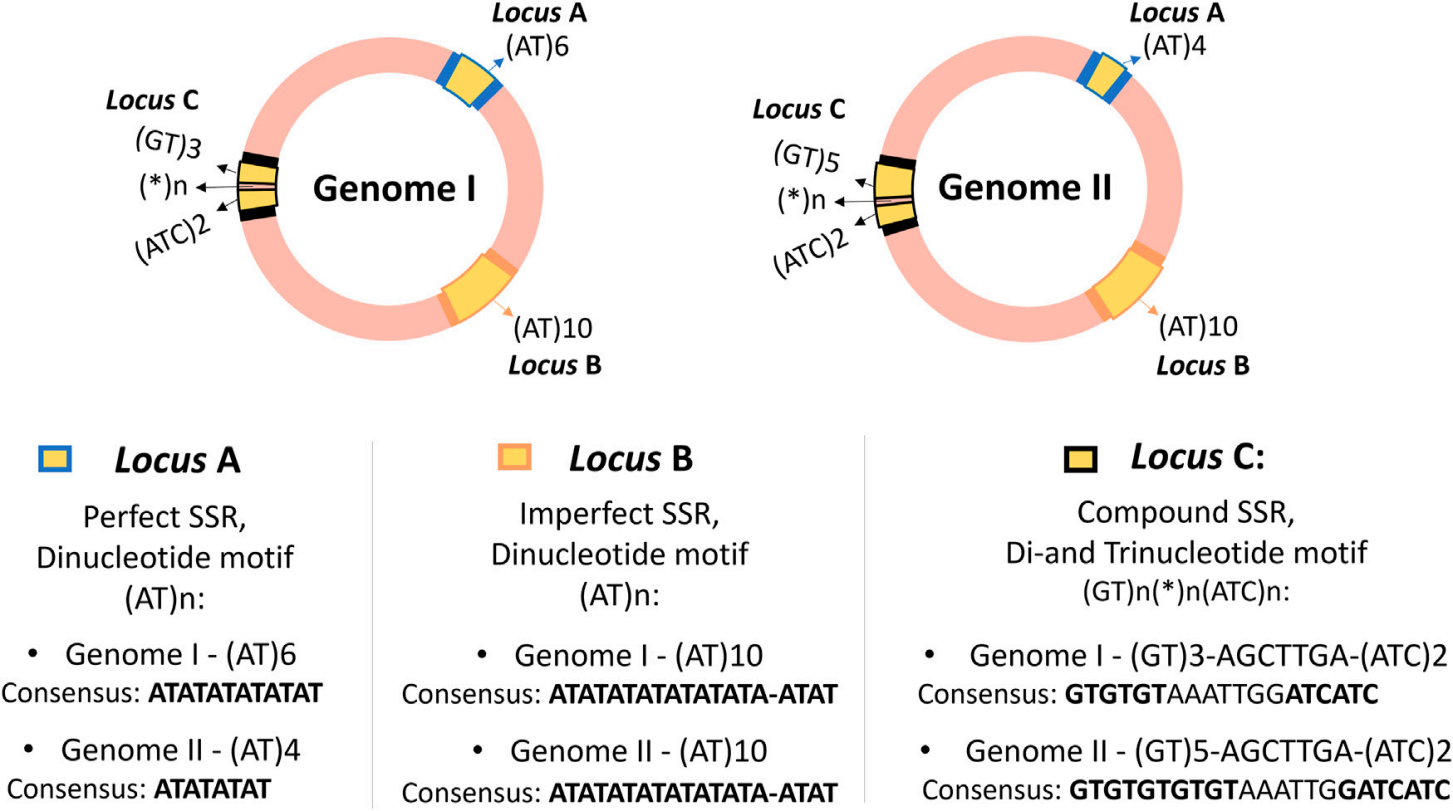
Representation of three SSR loci in two hypothetical circular genomes of closely related prokaryotes, showing the distribution of SSR loci, their flanking regions, classification, and consensus sequence of the loci. The genomes are highlighted in pink, with SSR loci in yellow (loci A, B, and C) and flanking regions in blue, orange, and black. Although SSRs are also present in eukaryotic genomes, they are didactically illustrated as prokaryotic circular genomes for easier visualization of locus positions. The loci are present in both genomes, suggesting they could be molecular marker candidates. The same motifs can appear in different regions, as seen with loci A and B with the motif (AT)n, but they are considered distinct SSR loci based on flanking regions and genetic context, not just the repeat motif. This example also illustrates possible variations without generalizing polymorphism patterns, serving only to demonstrate examples of Perfect, Imperfect, and Compound SSR loci. Loci A and C show length polymorphism, while locus B shows no length polymorphism, despite presenting a conserved deletion in one of its repeats.

Microsatellites are short repetitions in tandem of motifs consisting of 1–6 base pair (bp), that may appear with or without interruptions, distributed across the genomes of all known organisms, including eukaryotes, prokaryotes, and viruses, as well as in some organelles (Chambers and MacAvoy, 2000; Sahu et al., 2020). A single genome can contain thousands of distinct microsatellite loci, as illustrated in Figure 1, where three SSR loci were compared in two circular genomes of prokaryotes. SSR loci are particularly useful in fields where the polymorphism of specific loci can be compared and analyzed to establish close relationships, such as in paternity testing in forensics (Vieira et al., 2016).

To truly understand what microsatellites are, however, this section aims to go beyond this traditional definition. It is anticipated that Figure 1 may prompt questions in the reader’s mind, as they may not yet be familiar with the specific terminology. To fully comprehend the advanced concepts illustrated in Figure 1, it is advisable to first understand that SSRs are polymorphic repetitive elements and to grasp their importance and classifications. Readers are encouraged to revisit this figure after reading this section. This deeper understanding will allow not only a clearer interpretation of SSRs but also aid in making sense of the outputs from SSR tools and extracting biological meaning from the computational predictions.

### 2.1 Repetitive elements, tandem repeats and microsatellites

**FIGURE 2:**
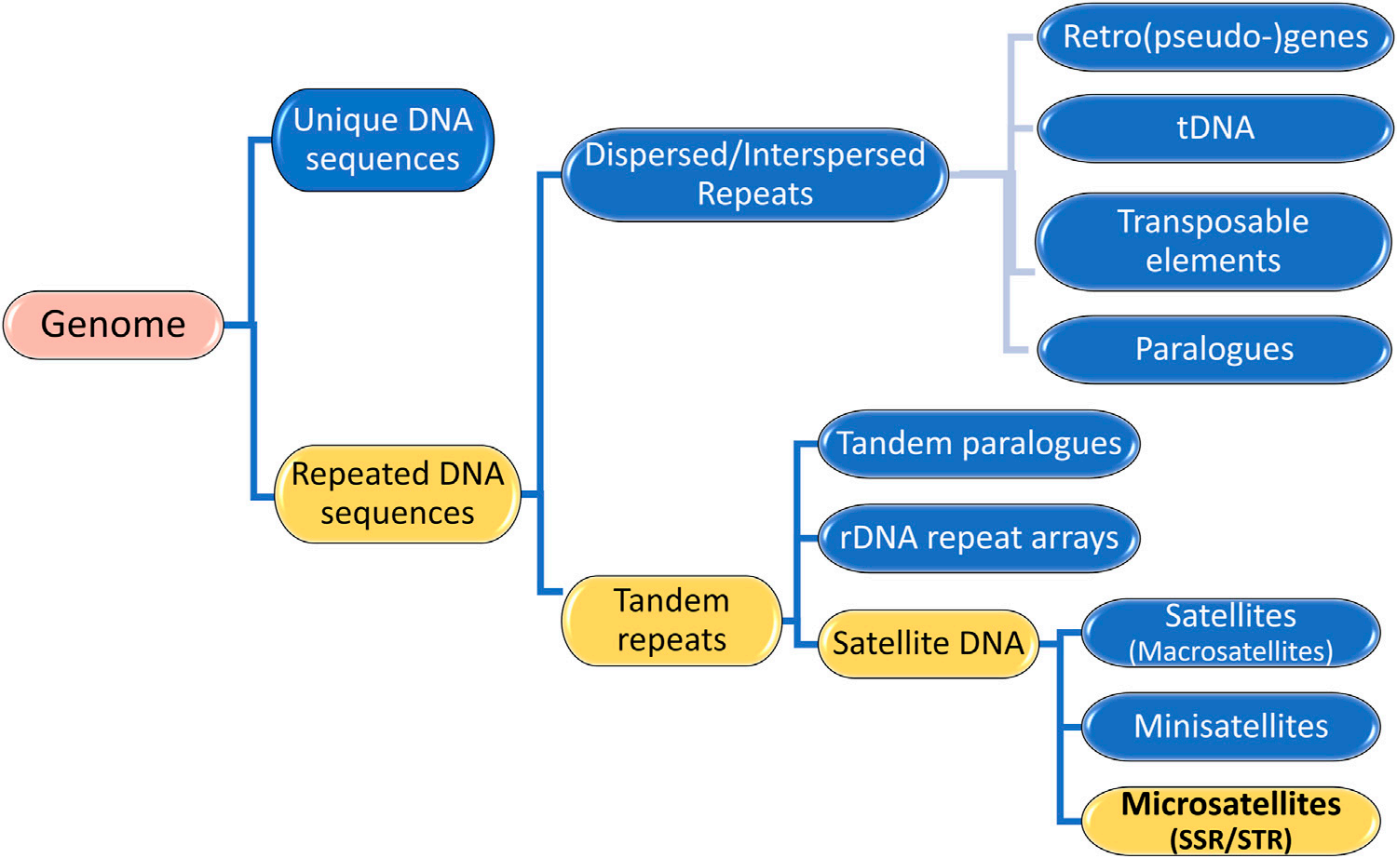
Genome Composition: Unique DNA sequences and Repeated DNA sequences. The illustration highlights that Microsatellites are Satellite DNA, a subcategory of Tandem Repeats, which are Repetitive Elements in a Genome.

Genomes consist of numerous DNA sequences organized into arrays of different sizes. As depicted in Figure 2, a continuous segment of DNA is called a sequence and is categorized into (i) unique DNA sequences and (ii) repeated DNA sequences, also known as Repetitive Elements or repeats (Richard et al., 2008). Unique segments are non-repetitive, while repetitive elements appear multiple times in the genome (Agarwal and States, 1994; Chambers and MacAvoy, 2000; Richard et al., 2008; da Silva Lopes et al., 2015). Repetitive Elements (RE) can be classified into two groups: (i) Dispersed or Interspersed repeats and (ii) Tandem repeats (Figure 2) (Richard et al., 2008; Lerat, 2010; Girgis and Sheetlin, 2013; Dumbovic et al., 2017; Srivastava et al., 2019).

**FIGURE 3:**
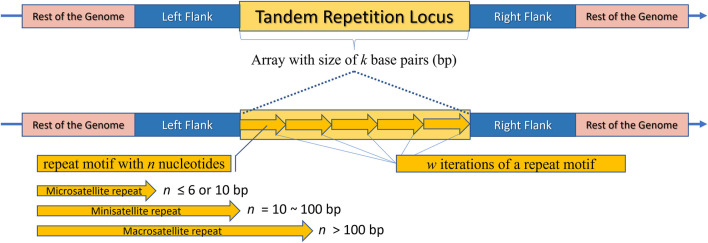
Schematic representation of a Tandem Repeat locus structure. The rest of the genome is highlighted in pink, flanking regions in blue, and the repeat locus itself in yellow. The locus consists of an array with a size of “k” base pairs. The bottom part of the image shows an enlarged view of the locus, where arrows are repeated side by side “w” times, representing the tandemly repeated motifs. It is noteworthy that each arrow represents an illustrative motif, which is a pattern composed of “n” nucleotides. Depending on “n”, the locus can be classified as a Microsatellite, Minisatellite, or Macrosatellite.

Tandem repeats (TR) comprise repetitive sequences that occur head-to-tail arrangement and their classification includes (i) gene tandems, (ii) ribosomal DNA (rDNA) repeat arrays, and (iii) satellite DNA (Figure 2) (Richard et al., 2008; Lerat, 2010). A tandem repetition locus is an array with size of “k” base pairs (bp), consisting of “w” iterations of a repeat motif with “n” nucleotides, being mutable regions flanked by sequences that are usually conserved (Figure 3) (Alves et al., 2023). Satellite DNA, a type of tandem repeat, can be further classified as (i) satellite or macrosatellite, (ii) minisatellite or (iii) microsatellite (Figure 2) (Lim et al., 2013; Avvaru et al., 2018). As illustrated in Figure 3, this classification is based on the size of the repeated nucleotide pattern, designated as “n” or “motif”. If the repeat motif has large periods, exceeding 100 bp, it is named macrosatellite; those with periods exceeding 10 bp are known as minisatellites, while microsatellites are short tandem repeats with motifs of “n” ≤ 6 bp (Buschiazzo and Gemmell, 2006; Kelkar et al., 2010; Chen et al., 2011a; Lim et al., 2013; Saeed et al., 2016; Dumbovic et al., 2017; Guang et al., 2019; Alves et al., 2023).

Nevertheless, there is no consensus regarding the classification of microsatellites and minisatellites, which has led to distinct categorizations among researchers (Chambers and MacAvoy, 2000; Lim et al., 2013; Alves et al., 2023). While most papers define SSRs as 1 to 6 base pair repeats, some consider larger motifs up to 10 bp still as SSR. This results in a microsatellite threshold ranging from 6 to 10 bp (Richard et al., 2008; Churbanov et al., 2012; Korotkov et al., 2021). The terminology surrounding the variable number of tandem repeats (VNTRs) also faces challenges in terms of consensus. Some authors classify these sequences as synonymous with minisatellites, while other researchers categorize them within the broader group of microsatellites and minisatellites (Karaca et al., 2005; Zribi et al., 2016; Lu et al., 2021).

The repetitive nature of these regions often results in sequencing errors, misalignment, and incomplete assemblies, especially when using short-read sequencing. This is a critical limitation, as many pathogenic STR alleles are longer than short reads (Uguen et al., 2024). Frequently, this leads to the absence of these regions from reference genomes or their misplacement within the genomic context. However, despite once being regarded as non-functional or “junk” DNA, numerous repeats have since been identified as important structural or evolutionary markers (Dumbovic et al., 2017). Recent advances in long-read sequencing and alignment tools have enhanced SSR detection by capturing full repeat regions, overcoming some limitations inherent to short-read (Editorial Nature, 2024).

### 2.2 Importance of microsatellites

SSR loci are highly polymorphic, prone to genetic mutations due to errors in DNA replication, recombination, or defective mismatch repair (dMMR), leading to microsatellite instability (MSI) (Yamamoto et al., 2024). This instability can result in the addition or deletion of SSR motifs (Figure 1), leading to length polymorphism and the generation of new inheritable SSR alleles (Jäger, 2022; Mokhtar et al., 2023). An allele with a frequency exceeding 1% within a population is considered polymorphic (Xia et al., 2016; Das et al., 2019; Guang et al., 2019).

Due to their significant role in genetic variation, SSRs have emerged as valuable molecular markers for genetic analysis. Their high variability and polymorphism, along with co-dominant inheritance and non-random distribution in the genome, contribute to their utility (Palliyarakkal et al., 2011). Moreover, the reproducibility of SSRs and the specific design of primers facilitate their amplification (Untergasser et al., 2012), enabling differentiation within and between populations (Marwal et al., 2014; Alves et al., 2023). Researchers explore various aspects of MS, including their incidence, frequency, prevalence, abundance, distribution, polymorphisms, composition, information content, localization, transferability, and associations with other sequence elements (Thiel et al., 2003; Sharma et al., 2007; Jilani and Ali, 2022).

SSRs widespread presence allows for comprehensive studies of DNA, transcribed sequences, and their corresponding proteins, as they can be found in both coding and non-coding regions, and are identifiable in various contexts, including sequenced DNA, assembled genomes, genes, and expressed sequence tags (ESTs) (Thiel et al., 2003; Karaca et al., 2005; Mathur et al., 2020).

In addition, MS have diverse applications across various fields. They are associated with over 30 human genetic diseases (Marwal et al., 2014; Lu et al., 2021) and very important in oncology (Baudrin et al., 2018). For instance, Indels in coding microsatellites (cMS) within tumor suppressor genes like *TGFBR2* and *ACVR2* act as key drivers of cancer progression in mismatch repair-deficient (MMRd) cells, generating immunogenic frameshift peptide (FSP) neoantigens. Darwinian selection favors cMS mutations that enhance cell survival and tumor growth, resulting in their accumulation. Thus, MMRd cancers are immunogenic not only due to a high number of somatic mutations but also the abundance of FSP-derived epitopes generated by these indels (Hernandez-Sanchez et al., 2022).

In forensics, STRs can be used for identification and parentage determination, as they form a “genetic fingerprint” for each individual (Haddrill, 2021; Jäger, 2022). By analyzing the distribution of specific STR alleles across populations, researchers can uncover group relationships and trace migration patterns, providing insights into human evolutionary history (Editorial Nature, 2024). Moreover, MS have been linked to influencing virulence in pathogens (Reneker et al., 2004) and can serve as biomarkers in fungi (Sokolova et al., 2022), protozoa (Durigan et al., 2018), bacteria (da Costa Pinheiro et al., 2022), and viruses (Laskar et al., 2022). They can also be applied in diagnostics, as exemplified by the investigation of leprosy transmission utilizing microsatellite typing through amplification of compound SSR loci (Mohanty et al., 2019).

Furthermore, SSRs are widely applied in plant research and breeding, providing insights into genetic diversity, population structure, and evolutionary patterns (Biswas et al., 2018). They aid in crop improvement by identifying alleles linked to desirable traits (Oliveira et al., 2008), and can play a crucial role in conservation biology by assessing genetic diversity in endangered plants (Yuan et al., 2018). Additionally, SSRs help trace the evolutionary history of plant species, providing insights into their adaptation and divergence (Morgante et al., 2002; Oliveira et al., 2008; Yuan et al., 2018).

### 2.3 Classifications of microsatellites

**FIGURE 4:**
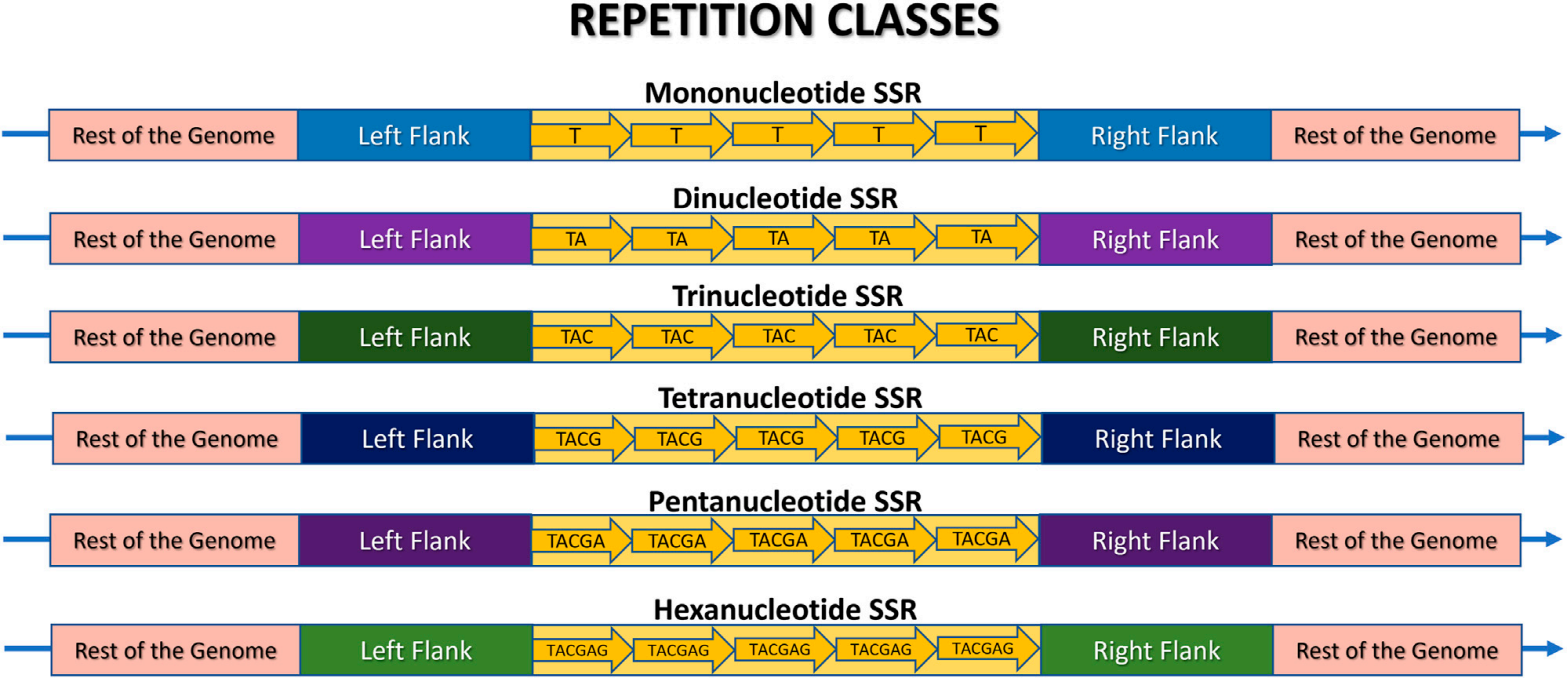
Schematic representation of a Microsatellite locus structure and its various repeat classes: mono-, di-, tri-, tetra-, penta-, and hexanucleotide sequences. The rest of the genome is highlighted in pink, the flanking regions are in different colors to indicate their presence in the same genome, but at different positions, and the SSR locus itself is in yellow. Each arrow represents an illustrative motif, which is a pattern composed of “n” nucleotides iterated “w” times. In this example, w = 5 for didactic comparison purposes. Thus, the illustrative mononucleotide (n = 1) is represented as (T)5, the dinucleotide (n = 2) as (TA)5, the trinucleotide (n = 3) as (TAC)5, the tetranucleotide (n = 4) as (TACG)5, the pentanucleotide (n = 5) as (TACGA)5, and the hexanucleotide (n = 6) as (TACGAG)5.

**FIGURE 5:**
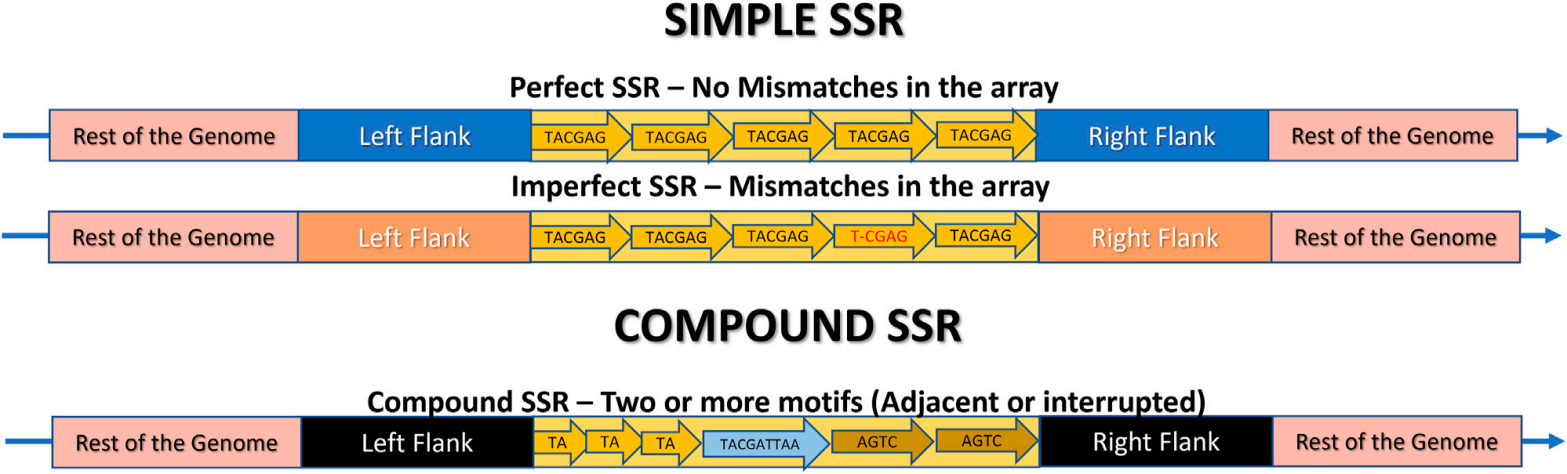
Schematic representation of a Microsatellite locus structure and its classification as Simple (Perfect, Imperfect) or Compound. The rest of the genome is highlighted in pink, the flanking regions are in different colors to indicate their presence in the same genome, but at different positions, and the SSR locus itself is in yellow. The arrow indicates the repeats. In Perfect SSRs, all repeat motifs are identical. In imperfect SSRs, almost all motifs are identical, but there are mismatches in one or more of them, highlighted in red. In compound repeats, there are two or more motifs composing the same SSR, which can be perfect or imperfect, and can be adjacent (side by side) or interrupted, meaning they are separated by a maximum distance.

In the literature it is usual to see SSR categorized based on various parameters (Figure 1). Common classifications include those based on repeat classes, perfection level, and tract composition, as illustrated in Figures 4, 5. Additional sub-classifications may be applied depending on specific research objectives (Mudunuri and Nagarajaram, 2007; Chen et al., 2011b; Ledenyova et al., 2019; da Costa Pinheiro et al., 2022).

#### 2.3.1 Based on repetition classes

Microsatellites can be classified into repetition classes based on the size “n” of their repeated nucleotide pattern, which is commonly referred to as the motif (Figure 3). As illustrated in Figure 4, the repetition classes of SSRs include: (i) Mononucleotide, which consists of “w” repetitions of a single nucleotide (n = 1), for example, (T)5; (ii) Dinucleotide, composed of “w” repetitions of a pair of nucleotides (n = 2), such as (TA)9; (iii) Trinucleotide, characterized by “w” repetitions of three nucleotides (n = 3), such as (TAC)12; (iv) Tetranucleotide, consisting of “w” repetitions of four nucleotides (n = 4), e.g., (TACG)8; (v) Pentanucleotide, formed by “w” repetitions of five nucleotides (n = 5), for instance (TACGA)4; and (vi) Hexanucleotide, consisting of “w” repetitions of six nucleotides (n = 6), such as (TACGAG)5 (Sharma et al., 2007; Sahu et al., 2020).

Usually, SSR tools permit users to define the minimum number of repetitions (*w*) that must occur for a motif (*n*) to be considered an SSR. In instances where the tool identifies a repetition of the motif with fewer than the specified repetitions, that SSR will not be included in the final output (Alves et al., 2023). It is worth noting that there is no standard value for this parameter, as it can vary depending on the specific organism under study. Therefore, it is advisable for users to consult relevant literature on the organism in question to identify the most suitable parameters for their study (Oliveira et al., 2008).

#### 2.3.2 Based on perfection level

As SSRs are prone to suffer mutations, they can be classified as (i) Perfect SSR (p-SSR or pSSR) or (ii) Imperfect SSR (i-SSR or iSSR) (Figure 5) (Mokhtar and Atia, 2019; Song et al., 2021).

Perfect microsatellite tracts, also referred as Pure or Exact, consist of motifs that are replicated multiple times with precise replication of the exact pattern, such as (AT)20 and the examples in Figure 4, without any deviations. However, as mutational events occur, mismatches can arise, leading to disruptions in the p-SSR through base substitutions or nucleotide insertions or deletions (INDELs) (Figure 5). Consequently, the p-SSR is transformed into an imperfect SSR when the copies deviate by at least one base pair. The i-SSR may also be referred to as Approximate or Interrupted (Sharma et al., 2007; Lim et al., 2013; da Costa Pinheiro et al., 2022). For example, if the p-SSR (AT) 20 was interrupted due to an insertion of a “G” it would be classified as an i-SSR (AT) 12 G (AT) 8 by some SSR mining tools (Alves et al., 2023).

Most research in this field has concentrated on perfect repeats, given their association with selective forces and higher length polymorphism. Consequently, the prevalence of SSR tools with algorithms that can only identify perfect SSR (Mudunuri and Nagarajaram, 2007; Behura and Severson, 2015; Ledenyova et al., 2019). In contrast, imperfect SSRs are more stable and less susceptible to slippage mutations, resulting in less length polymorphism but featuring INDELs, which can be valuable for studying single nucleotide polymorphisms (SNPs) (Metz et al., 2016; Guang et al., 2019). It is worth noting that when mismatches are allowed in SSR tools, some SSRs previously identified as p-SSR may be elongated and reclassified as i-SSR arrays (Alves et al., 2023).

#### 2.3.3 Based on tract composition

Microsatellite tracts may exhibit two distinct compositions. They may either comprise a single motif or a combination of motifs. In this way they can be categorized as (i) Simple SSRs or (ii) Compound SSRs (c-SSRs, cSSRs) (Figure 5) (George et al., 2015; Sahu et al., 2020; Song et al., 2021).

Simple SSRs comprise p-SSR and i-SSR and consist of loci with a unique motif repeated in tandem, such as (TA)7. Compound microsatellites, also referred as Fuzzy or Interrupted by authors, arise from mutations or imperfections in SSR (George et al., 2015). They are loci composed of two or more simple SSR motifs, that might be adjacent or interrupted, separated by a nucleotide sequence (Chen et al., 2011b; Ledenyova et al., 2019). For instance, in Figure 5, “X” represents the sequence interrupting the two parts of the SSR in (TA)3-X-(AGTC)2. In Figure 1 simple and compound SSR loci are illustrated and compared in two hypothetic circular genomes.

In the context of SSR mining tools, whether two simple microsatellites are regarded as a c-SSR is contingent upon the distance between motifs resulting from the interrupting sequence. For instance, two SSRs separated by distances falling within a specified range (dMAX) may be regarded as a single c-SSR tract. However, depending on the dMAX set by the user, it is possible that some C-SSRs could be considered as two distinct SSRs (Alam et al., 2019).

#### 2.3.4 Based on genomic context

Microsatellites can be classified as either (i) Coding SSRs, if they are fully or partially situated within coding regions, or (ii) Non-coding SSRs, if they are located in regions that do not encode proteins (Mudunuri and Nagarajaram, 2007; da Costa Pinheiro et al., 2022; Alves et al., 2023).

The presence of SSRs in coding regions results in the emergence of these repetitive patterns in transcribed sequences and in their proteins, reflecting potential associations with genes and phenotypes (Vieira et al., 2016).

In general, the most SSRs are present at intergenic and non-coding regions, and less frequent in exons and genic regions (Srivastava et al., 2019). This is primarily attributed to the high mutation rate of microsatellites, which could potentially disrupt gene expression (Vieira et al., 2016). Most coding regions are composed of SSRs with tri- and hexanucleotide motifs. This is likely due to the selective pressure against mutations that could alter the reading frame (Li et al., 2002; Vieira et al., 2016). The length variations of SSRs within exons have been associated with various diseases, including Huntington’s and Spinocerebellar Ataxia (Srivastava et al., 2019).

Some SSR mining tools can ascertain whether an SSR resides in a coding region, although this requires the input of an additional file by the user, in which the regions that are and are not genetic should be indicated (Alves et al., 2023).

#### 2.3.5 Based on mutability and array length

Regarding their mutability, microsatellites can be categorized as: (i) Hypermutable SSRs, which consist of multiple repeat units that exhibit high rates of INDELs; (ii) Mutable SSRs, which have intermediate-length repeat tracts and therefore lower mutation rates; and (iii) Proto-mutable SSRs, which consist of a small number of repeat units and exhibit mutation rates slightly higher than the average for the genome (Bidmos and Bayliss, 2014).

There is an additional classification that considers mutability and array length, designated “k” This classification is as follows: (i) Class I, hypervariable markers, with arrays exceeding 20 base pairs (bp); (ii) Class II, potentially variable markers, with arrays from 12 to 20 bp; and (iii) Class III, SSRs, less variable markers, characterized by smaller arrays with less than 11 bp (Temnykh et al., 2001; Saeed et al., 2016).

Although the classifications in question might not be commonly employed in a general context, they do exist in certain tools designed to study polymorphic SSRs (PolySSRs). Consequently, this classification should be carefully considered, particularly by those engaged in the development of polymorphic SSR markers (Xia et al., 2016).

At this point, the reader has been introduced to the fundamental concepts and significance of microsatellites, which are crucial for understanding their applications in various fields. With this foundational knowledge, Figure 1 should now be more comprehensible, offering clarity on the discussed principles.

## 3 Section 2: a guide to tools for identifying microsatellites

The standard procedure for *in silico* SSR analysis involves obtaining the DNA data to be analyzed and using it as input for an SSR tool. These tools typically identify SSRs and their positions in the sequence, enabling various analyses. However, the abundance of available tools may challenge users in identifying the most suitable one for *their in silico* studies (Alves et al., 2023). Considering that the reader now possesses understanding of the key concepts of MS, this section aims to provide a comprehensive analysis of the tools utilized for tandem repeat prospecting, focusing on microsatellite mining software, and provide guidance on selecting the most appropriate tool for their specific research needs.

To achieve this, a literature review was conducted on Pubmed with the terms “Review” and “SSR” or “STR” or “Microsatellites”, and after careful evaluation were included in the study all review papers that focused on tools for SSR mining. To find more tools, this was complemented by a comprehensive survey of the broader SSR literature, with particular attention to references made in papers that released new softwares. These articles often compare the newly launched tools with existing ones, enabling the identification of most tools released to date. A total of 74 tools were identified, with every SSR tool found included in the analysis. If a tool is not listed, it was likely not discovered by the authors at the time this paper was written. The citation index for each tool’s publication was obtained from Google Scholar, and citations were compared to determine which tools were most widely adopted by the scientific community. Their availability was assessed, and an in-depth analysis was conducted for each functional tool, including parameters, inputs, outputs, and other relevant aspects, which are discussed in the following sections.

### 3.1 Overview of all tools identified in this paper

#### 3.1.1 Previous reviews of microsatellite search tools

**FIGURE 6:**
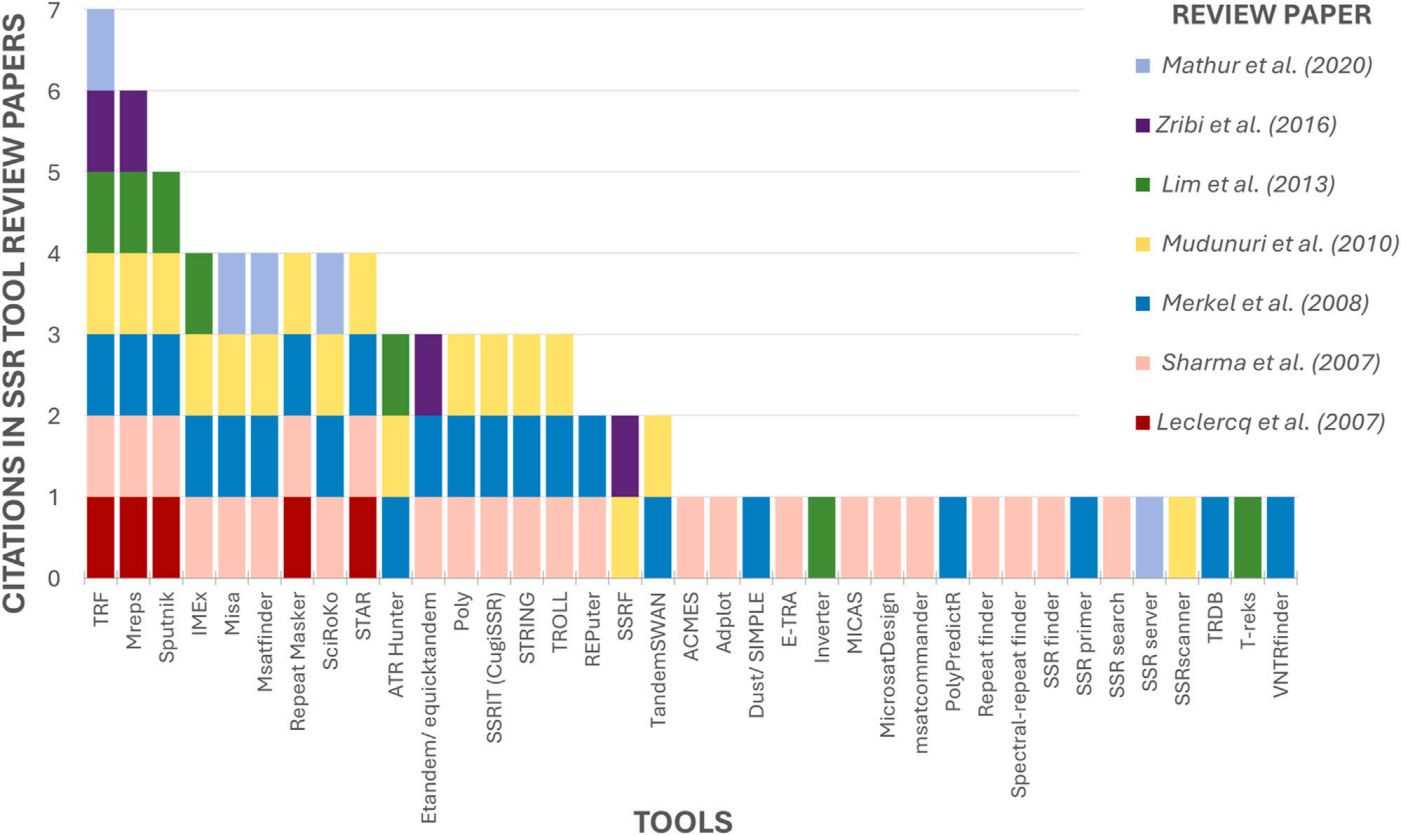
Ranking of mentioned tools by previous SSR tools Review papers. The X-axis contains the 37 tools mentioned in SSR tool review papers. The Y-axis indicates how many review papers cited each tool.

Seven reviews regarding SSR tools have been retrieved (Leclercq et al., 2007; Sharma et al., 2007; Merkel and Gemmell, 2008; Mudunuri et al., 2010b; Lim et al., 2013; Zribi et al., 2016; Mathur et al., 2020). The maximum number of tools mentioned in a single article was 25 (Sharma et al., 2007), and by combining the data from the authors, a total of 37 tools were identified, some of which were cited by most of the papers (Figure 6). The Tandem Repeats Finder (TRF) (Benson, 1999) stands out as the most frequently cited tool, having been cited by all the review papers. Second is Mreps (Kolpakov et al., 2003) mentioned in 06 papers, followed by Sputnik (La Rota et al., 2005), cited by 05 authors. Fourth on the list are IMEx (Mudunuri and Nagarajaram, 2007), Misa (Thiel et al., 2003) and STAR (Delgrange and Rivals, 2004), all of which are cited by the majority (04 out of 07 reviews). The remaining tools are mentioned in Tables 1, 2.

**TABLE 1:** Total tools identified part 1–42 tools available in 2024.

Name	Cited in reviews	Availability in 2024	Reference/Year	Citations	Tool’s link
BatchPrimer3	No	Yes	You 2008	961	http://wheat.pw.usda.gov/demos/BatchPrimer3/
CandiSSR	No	Yes	Xia 2016	56	https://github.com/xiaenhua/CandiSSR
Dot2Dot	No	Yes	Genovese 2019	15	https://github.com/Gege7177/Dot2dot
EasySSR	No	Yes	Alves 2023	1	https://computationalbiology.ufpa.br/easyssr/
Etandem/Equicktandem	Yes	Yes	Rice 2000	N/A	https://www.bioinformatics.nl/cgi-bin/emboss/help/equicktandem
FullSSR	No	Yes	Metz 2016	18	https://sourceforge.net/projects/fullssr/
GMATa	No	Yes	Wang 2016	211	https://sourceforge.net/projects/gmata/?source=navbar
GMATo	No	Yes	Wang 2013	119	https://sourceforge.net/projects/gmato/files/?source=navbar
IDSSR	No	Yes	Guang 2019	15	https://github.com/Allsummerking/IDSSR
IMEx	Yes	Yes	Mudunuri 2007	231	http://www.mcr.org.in/IMEX/download.html
Imperfect SSR Finder	No	Yes	Stieneke 2007	10	https://ssr.nwisrl.ars.usda.gov/
Kmer-SSR	No	Yes	Pickett 2017	25	https://github.com/ridgelab/Kmer-SSR
Krait	No	Yes	Du 2018	132	https://github.com/lmdu/krait
MegaSSR	No	Yes	Mokhtar 2023	1	https://bioinformatics.um6p.ma/MegaSSR
MICAS	Yes	Yes	Sreenu 2003	27	http://www.mcr.org.in/micas/
Micro-Primers	No	Yes	Alves 2022	2	https://github.com/FilAlves/micro-primers
Microsatellite repeats finder	No	Yes	Bikandi 2006	5	http://insilico.ehu.es/mini_tools/microsatellites/?info
MiMi	No	Yes	Fox 2019	16	https://github.com/graemefox/mimi
MISA	Yes	Yes	Thiel 2003	2602	https://webblast.ipk-gatersleben.de/misa/
Mreps	Yes	Yes	Kolpakov 2003	477	https://mreps.univ-mlv.fr/
msatcommander	Yes	Yes	Faircloth 2008	1071	https://code.google.com/archive/p/msatcommander/
PALfinder	No	Yes	Castoe 2012	300	https://sourceforge.net/projects/palfinder/
PERF	No	Yes	Avvaru 2018	41	https://github.com/rkmlab/perf
PolyMorphPredict	No	Yes	Das 2019	17	http://webtom.cabgrid.res.in/polypred/
PolySSR	No	Yes	Tang 2008	102	http://www.bioinformatics.nl/tools/polyssr/
RepeatMasker	Yes	Yes	Smit 1996 apud Tarailo-Graovac 2009	2443	https://www.repeatmasker.org/cgi-bin/WEBRepeatMasker
REPuter	Yes	Yes	Kurtz 2001	1859	http://bibiserv.techfak.uni-bielefeld.de/reputer/
SA-SSR	No	Yes	Pickett 2016	15	https://github.com/ridgelab/SA-SSR
SciRoKo	Yes	Yes	Kofler 2007	399	https://kofler.or.at/bioinformatics/SciRoKo/index.html
Spectral-repeat finder	Yes	Yes	Sharma 2004	196	http://www.imtech.res.in/raghava/srf
Sputnik	Yes	Yes	Morgante 2002	1465	http://wheat.pw.usda.gov/ITMI/EST-SSR/LaRota/
SSR_pipeline	No	Yes	Miller 2013	57	https://pubs.usgs.gov/ds/778/
SSR2Marker	No	Yes	Yue 2022	1	https://github.com/aaranyue/SSR2Marker
SSRenricher	No	Yes	Luo 2020	3	https://github.com/byemaxx/SSREnricher
SSRIT (CugiSSR)	Yes	Yes	Temnykh 2001	2056	https://archive.gramene.org/db/markers/ssrtool
SSRMMD	No	Yes	Gou 2020	25	https://github.com/GouXiangJian/SSRMMD
SSRpoly	No	Yes	Duran 2013	8	https://appliedbioinformatics.com.au/Edwards/index.php/SSRPoly
STAR	Yes	Yes	Delgrange 2004	142	http://atgc.lirmm.fr/star/
T-reks	Yes	Yes	Jorda 2009	196	https://bioinfo.crbm.cnrs.fr/index.php?route=tools&tool=3
TRF	Yes	Yes	Benson 1999	7903	https://tandem.bu.edu/trf/trf.html
TROLL	Yes	Yes	Castelo 2002	265	https://sourceforge.net/projects/finder/
Websat (WebTROLL)	No	Yes	Martins 2009	365	https://bioinfo.inf.ufg.br/websat/

“Yes” has been highlighted in the columns for visualization purposes.

**TABLE 2:** Total tools identified part 2–32 tools not available in 2024.

Name	Cited in reviews	Availability in 2024	Reference/Year	Citations	Tool’s link
ACMES	Yes	No	Reneker 2004	16	http://acmes.rnet.missouri.edu/
Adplot	Yes	No	Taneda 2004	25	Email
ATR Hunter	Yes	No	Wexler 2004	141	http://www.bioinfo.cs.technion.ac.il/ATRHunter
Dust/SIMPLE	Yes	No	Hancock 1994	170	http://life.anu.edu.au/
E-TRA	Yes	No	Karaca 2005	51	ftp.akdeniz.edu.tr/Araclar/e-TRA
Inverter	Yes	No	Wirawan 2010	14	http://bmserver.sce.ntu.edu.sg/INVERTER
LSAT	No	No	Biswas 2018	3	http://210.110.86.160/Lsat/Lsat.html
MfSAT	No	No	Chen 2011	3	http://hudacm11.mysinamail.com/hunan.html
MicrosatDesign	Yes	No	Singan 2005	4	http://daphnia.cgb.indiana.edu/wfleabase/software
Msatfinder	Yes	No	Thurston 2005	123	http://www.genomics.ceh.ac.uk/msatfinder/
Poly	Yes	No	Bizzaro 2003	36	http://bioinformatics.org/poly/wiki/
PolyPredictR	Yes	No	Odushlaine 2006	16	http://bioinformatics.rcsi.ie/vntrfinder/
ProGeRF	No	No	Lopes 2015	11	http://64.79.105.19/ligp/
QDD	No	No	Meglecz 2010	694	http://www.univ-provence.fr/gsite/Local/egee/dir/meglecz/QDD.html
RepeatFinder	Yes	No	Volfovsky 2001	203	http://www.tigr.org/softlab/
ReRep	No	No	Otto 2008	15	http://bioinfo.pdtis.fiocruz.br/ReRep/
Risa	No	No	Kim 2012	5	http://sol.kribb.re.kr/RISA/
SAT	No	No	Dereeper 2007	40	http://sat.cirad.fr/sat
SSRF	Yes	No	Sreenu 2003	27	Email
SSRfinder	Yes	No	Gao 2003	388	https://www.fresnostate.edu/ssrfinder/
SSRlocator	No	No	Da Maia 2008	276	http://microsatellite.org/ssr.php
SSRprimer	Yes	No	Jewell 2006	105	http://bioinformatics.pbcbasc.latrobe.edu.au/ssrdiscovery.html
SSR-Primer Generator	No	No	Hong 2011	1	http://168.188.15.158:8080/ssrpg/
SSRscanner	Yes	No	Anwar 2006	5	Email
SSRsearch	Yes	No	Nicot 2004	286	ftp://ftp.gramene.org/pub/gramene/software/scripts/ssr.pl
SSRserver	Yes	No	Jung 2007	267	https://www.rosaceae.org/node/55
STRING	Yes	No	Parisi 2003	79	http://www.caspur.it/∼castri/STRING/
TandemSWAN	Yes	No	Boeva 2006	115	http://bioinform.genetika.ru/projects/swan/www/
TRDB	Yes	No	Gelfand 2007	218	https://tandem.bu.edu/cgi-bin/trdb/trdb.exe
TReaDS	No	No	Pellegrini 2012	14	http://bioalgo.iit.cnr.it/treads/
TRStalker	No	No	Pellegrini 2010	40	http://bioalgo.iit.cnr.it/
VNTRfinder	Yes	No	Odushlaine 2006	16	http://bioinformatics.rcsi.ie/vntrfinder/

“Yes” has been highlighted in the columns for visualization purposes.

Only 18 tools, approximately 49% of the total, were cited by more than one author (Figure 6), indicating that despite the numerous tools that exist, previous review articles have been limited to examining only a few of them. This shows that the current state of knowledge is scattered and that a comprehensive review such as the present study is needed to collect and analyze the maximum possible number of tools.

In addition to the 37 tools referenced in previous reviews, an exhaustive examination of microsatellite literature and an analysis of references cited in other published tools identified an additional 37 tools, resulting in a total of 74 tools that will be examined in the following sections. These tools were identified and summarized in Tables 1, 2, with further details provided in the subsequent sections.

#### 3.1.2 Analysis of availability of the tools

**FIGURE 7:**
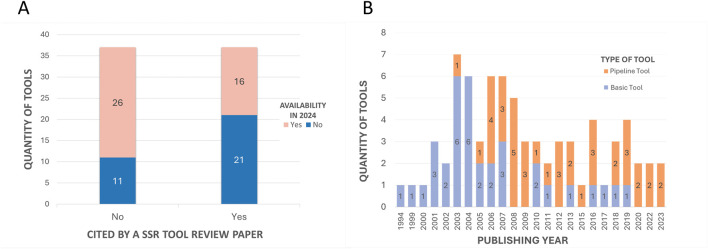
Quantitative comparisons with subclassifications. **(A)** - Comparison of the number of tools cited in previous SSR tool review articles and those cited exclusively in the current study, with subclassifications by the availability of tools. The “Yes” column on the right represents the 37 tools cited by the 07 previous review articles. The “No” column represents the other 37 tools mentioned in this article that were not mentioned in the other reviews. In both columns, the 42 tools that are available in 2024 (Table 1) are highlighted in pink, while the 32 that are no longer available (Table 2) are highlighted in blue. **(B)** - Comparison of the number of tools published per year with subclassifications by tool type. The subclassification includes basic tools (single-purpose tools that perform specific functionalities and/or serve as a base for others to integrate into the pipeline) or pipeline tools (tools that integrate several others).

The first aspect that was considered was the tool’s availability for present-day usage by users. Given the dynamic nature of the field, there is a risk that individuals relying on previous review articles to guide their tool selection for methodologies might find their chosen tool non-operational. All 74 tools were assessed regarding their accessibility status. Multiple attempts were made to access the tools between 2023 and 2024 using the links provided in the papers. Figure 7A illustrates that only half of the total 74 tools had previously been cited in reviews. Of these 37 tools referenced in the review articles, only 16 are still available. This implies that 57% of the previously reviewed tools are no longer functional. Conversely, 70% of the tools identified in this article alone are available. Overall, out of the total of 74 tools initially identified, 42 remained functional and were summarized in Table 1, while the 32 tools not accessible at the time of analysis were labeled as not available and grouped in Table 2.

#### 3.1.3 Analysis of temporality of tool’s release and citations

The historical development of microsatellite analysis can be traced through the papers of each tool (Tables 1, 2). These indicate a temporal range of release years between 1994 and 2023, as evidenced in Figure 7B. This suggests a dynamic evolution within the field, with the introduction of innovative tools and methodologies occurring continuously (Alves et al., 2022). Prior to 2005, the prevailing trend in the field was the development of basic tools that introduced new algorithms or approaches, performed specific functions, or served as a basis for integration into pipelines. From 2006 onwards, the focus has shifted to the release of pipeline tools, demonstrating a pattern where modern tools build upon established frameworks and incorporate additional features, to enhance analytical capabilities for various objectives.

The continuous release of new tools prompted an investigation into the relative prominence of these tools compared to older ones. The investigation also sought to determine whether the high citation counts for a tool could be attributed to the fact that it was mentioned in review articles. To test this hypothesis, the citation counts for each tool’s original papers were assessed using Google Scholar in early 2024 (Tables 1, 2).

**FIGURE 8:**
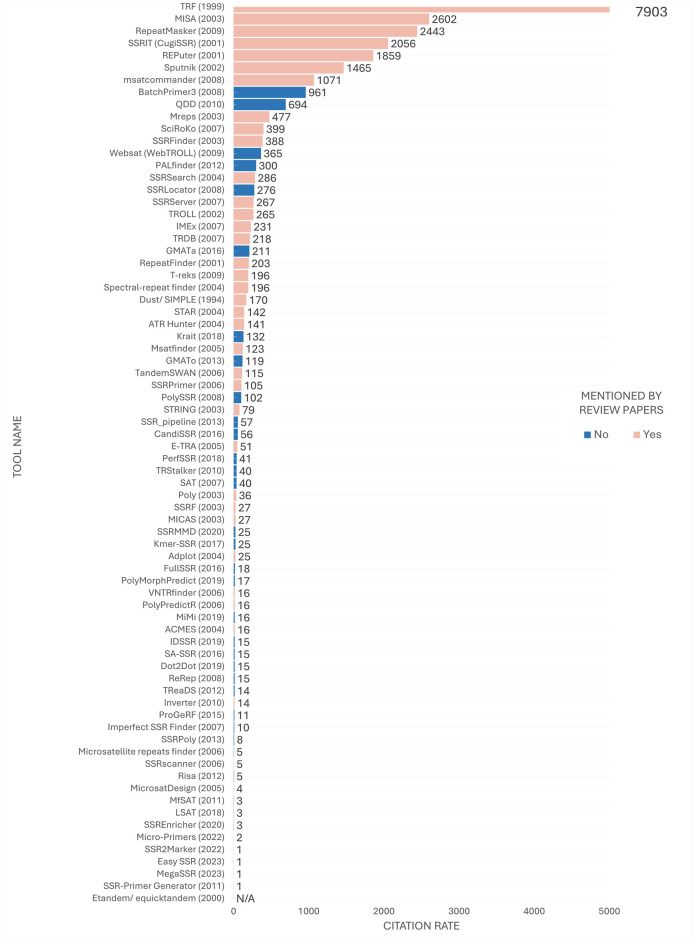
Citation ranking of SSR tools. Number of citations retrieved from Google Scholar in early 2024 by tool’s papers. The year of release is indicated in parenthesis next to the tool’s name for temporal comparison purposes. Tools mentioned in the review articles under study are highlighted in pink, while tools studied solely in the present study are highlighted in blue. The citation rate for the Etandem/equicktandem tools was considered N/A (Not Available) as their citations were combined with other tools citing EMBOSS, making it currently inviable to estimate the number of citations specifically for these tools.

A discrepancy was observed between the order of the tools most frequently mentioned in review articles (Figure 6) and their position in the citation ranking depicted in Figure 8. Nevertheless, a considerable number of the most frequently cited tools were referenced in review articles. At the time of analysis, TRF (Benson, 1999) remained the most frequently cited tool, with a substantial margin of citations over the second-ranked tool. Furthermore, some tools, despite being less frequently mentioned in review articles such as msatcommander (Faircloth, 2007), have received considerable citation counts. Conversely, the emergence of previously unmentioned tools with considerable citation indices, such as BatchPrimer3 (You et al., 2008) and GMATa (Wang and Wang, 2016), underscores the dynamic nature of the field.

Figure 8 also reveals that the top 20 most cited tools have remained in use for a minimum of a decade, indicating a concentration of citations in long-established tools over time. Conversely, despite their versatile features, more contemporary tools released in the last 5 years, such as EasySSR (Alves et al., 2023), MegaSSR (Mokhtar et al., 2023), Micro-primers (Alves et al., 2022), and SSR2Marker (Yue and Liu, 2022), appear not to have yet received substantial attention from the scientific community.

Although the tools on the top concentrate an impressive number of citations (Figure 8), this does not necessarily indicate that they are superior to others in the middle to bottom part of the citation ranking. If this were the case, there would be no incentive to develop new tools to address the limitations of existing ones (Sharma et al., 2007). Users often have specific expectations for tools that align with their research goals. While a suitable tool with the necessary features may exist, users may be unaware of it and therefore not use or cite it. Instead researchers may encounter difficulties in adapting their project to utilize a popular tool (Alves et al., 2023). Thus, the subsequent sections aim to disseminate knowledge regarding the tools, irrespective of the citation rate.

### 3.2 Comparison of the available tools

In the previous subsection, the availability status of every tool was identified, providing a clear understanding of which ones are operational. To help users concentrate on viable options, this section compares the 42 functional programs featured in the main paper, as summarized in Table 1. However, although they were not included in the main text discussion, the 32 non-functional programs (listed in Table 2) were also evaluated and their data is available in Supplementary Table S1, acknowledging the possibility that some may become operational again in the future. The complete gathered data for all 74 tools is also provided in Supplementary Table S1, which includes not only the analyzed information but also filters to facilitate deeper analysis, along with more specific data for each tool reviewed, such as the parameters utilized, input and output formats, features, and availability of each tool. It is advisable that users download the Supplementary Table S1 and take it as a resource to consult while selecting the tool to use in their research.

To facilitate comparison, the tools were summarized based on their focus of analysis–Repetitive Elements (RE), Tandem Repeats (TR), and Short Tandem Repeats (STR). These categories were further divided by tool type (Basic or Pipeline Tool) and by their ability to identify only perfect SSRs (p-SSR) or both perfect and imperfect SSRs (i-SSR). The main data was presented in tables to enhance user access to the comparison of specific features, accompanied by a brief analysis of select attributes, including advantages, disadvantages, and the applicability of each tool to SSR research. The tools were analyzed and discussed in alphabetical order, without bias toward citations. However, if readers wish to consider citation frequency as a criterion, they can refer to Figures 6, 8 to identify the most popular tools in the literature. Additionally, advanced users with specific research questions or familiarity with SSR tools can focus primarily on the tables in the text and Supplementary Table S1 to compare relevant aspects. Users may also filter for suitable tools, in the tables and Supplementary Table S1, then compare and read the descriptions of those selected tools, rather than reviewing all descriptions, as many tools share similar functionalities.

#### 3.2.1 Tools for detecting repetitive elements and tandem repeats

The analysis identified nine tools with a generalist focus, comprising three tools for detecting RE and six tools for TR (Table 3). As illustrated in Figure 2, short tandem repeats (STRs) are a type of tandem repeat, which belongs to the broader category of repetitive elements. These tools were included because, although STR are not their sole focus, they represent a significant subgroup within the broader spectrum of repetitive elements and tandem repeats, thus are present in their outputs (Sharma et al., 2007). Many studies and reviews have applied these tools for STR analysis, given that they usually include parameters that can be personalized for identifying MS (Gao et al., 2003; Leclercq et al., 2007). Additionally, it is essential to highlight that their citation rates observed in Table 1 and Figure 8 may not exclusively reflect citations in SSR-related projects, as these tools can also detect other types of repeats.

**TABLE 3:** Tools for detecting repetitive elements (RE) and tandem repeats (TR).

Name	Reference/Year	Type	Mining tool	Integrated toolset	Platform	Execution	Focus	Max motif lenght	Input files	Output graphs/Charts	p-SSR	i-SSR	c-SSR	Primer design	Tool’s link
RepeatMasker	Smit 1996 apud Tarailo-Graovac 2009	Pipeline Tool	RepeatMasker	cross_match, WU-BLAST	Linux, Web	Graphical Interface and Command Line	RE	No limit	FASTA	No	Yes	Yes	No	No	https://www.repeatmasker.org/cgi-bin/WEBRepeatMasker
REPuter	Kurtz 2001	Basic tool	REPuter	No	Linux, Mac OS, Web	Graphical Interface and Command Line	RE	<5 000 bp	DNA sequence	Yes	Yes	Yes	Yes	No	http://bibiserv.techfak.uni-bielefeld.de/reputer/
Spectral-repeat finder	Sharma 2004	Basic tool	Spectral-repeat finder	No	Linux, Mac OS, Windows, Web	Graphical Interface	RE	No limit	FASTA; GBK	No	Yes	Yes	No	No	http://www.imtech.res.in/raghava/srf
Dot2Dot	Genovese 2019	Basic tool	Dot2Dot	No	Linux, Mac OS	Command Line	TR	No limit	FASTA; FASTQ	No	Yes	Yes	No	No	https://github.com/Gege7177/Dot2dot
Etandem/Equicktandem	Rice 2000	Basic tool	Etandem/Equicktandem	No	Linux	Command Line	TR	1–600 bp	FASTA; GBK; GFF; EMBL and Others	No	Yes	No	No	No	https://www.bioinformatics.nl/cgi-bin/emboss/help/equicktandem
Mreps	Kolpakov 2003	Basic tool	Mreps	No	Linux, Windows, Web	Graphical Interface and Command Line	TR	No limit	FASTA	No	Yes	Yes	No	No	https://mreps.univ-mlv.fr/
STAR	Delgrange 2004	Basic tool	STAR	No	Linux, Mac OS, Windows, Web	Graphical Interface and Command Line	TR	No limit	FASTA	No	Yes	Yes	No	No	http://atgc.lirmm.fr/star/
T-reks	Jorda 2009	Pipeline Tool	T-reks	CLUSTALW, MUSCLE	Linux, Mac OS, Windows, Web	Graphical Interface and Command Line	TR	No limit	FASTA	No	Yes	Yes	No	No	https://bioinfo.crbm.cnrs.fr/index.php?route=tools&tool=3
TRF	Benson 1999	Basic tool	TRF	No	Linux, Mac OS, Windows, Web	Graphical Interface and Command Line	TR	1–5, 10–2000 bp	FASTA	No	Yes	Yes	No	No	https://tandem.bu.edu/trf/trf.html

“Yes” and “Pipeline” have been highlighted in the columns for visualization purposes. “RE” = repetitive elements, “TR” = Tandem Repeats. “BP” = Base pair. “p-SSR” = Perfect SSR. “i-SSR” = Imperfect SSR. “c-SSR” = Compound SSR.

Upon comparison of the tools, as depicted in Table 3, it was observed that none of them integrated primer design functionality natively. These tools employ a variety of algorithms, with some identifying only p-SSRs, both p-SSRs and i-SSRs, while only REPuter (Kurtz et al., 2001) allows the identification of compound SSRs and provides Graphs/Charts as outputs. Except for Dot2Dot (Genovese et al., 2019) and Etandem/Equicktandem (Rice et al., 2000), which can only be executed locally through the command line, the remaining tools offer a web server for graphical interface analysis. Dot2Dot stands out as the only tool that accepts FastQ files as input, while the others generally accept Fasta files.

##### 3.2.1.1 RE tools

RepeatMasker (Tarailo-Graovac and Chen, 2009) is a software developed by Smit and Green (A.F.A. Smit, R. Hubley and P. Green, unpublished data). It annotates repeats, replacing them with Ns, and returns masked sequences, a table of repeat content, and optional alignments. The web version has a 100 kb sequence limit, with longer sequences queued. It is important to note that RepeatMasker lacks specific parameters for defining the size of SSR motifs or the minimum number of iterations, offering options for selecting which repetitive elements to mask.

REPuter (Kurtz et al., 2001) offers comprehensive and efficient detection of various repeat types, coupled with a robust evaluation of their significance and interactive visualization capabilities. However, the online version of the software has a 5 Mb data size limit for uploaded data and a 5000-repeat cap due to server capacity constraints. For users who wish to analyze only SSRs, this program has limitations, as it only allows the definition of parameters such as minimal repeat size, maximum computed repeats, edit distance and hamming distance.

Spectral-repeat finder (SRF) (Sharma et al., 2004) employs an *ab initio* methodology, not relying on any prior assumptions regarding the length, fidelity, or arrangement of repeats. For users who seek to utilize RE tools for the study of SSR, this tool offers an appropriate level of flexibility, with a comprehensive range of parameters accessible via their web server, enabling users to identify repeats with varying sizes and specify a minimum number of repetitions. However, it should be noted that the web tool has a limitation in that it can only process one file at a time. Consequently, for larger projects, it is necessary to use a multifasta file or to perform individual genome analysis.

##### 3.2.1.2 TR tools

Dot2Dot (Genovese et al., 2019) employs a distinctive methodology for the identification of pure and fuzzy TRs through dot-plot matrices. As the only tool in this group to accept both assembled genomes (FASTA) and NGS data (FASTQ) as input, it allows the analysis of NGS sequences and generates tabular outputs in the formats “.bed” and “.dot”. While capable of identifying TRs of any size, this tool may be particularly valuable for researchers studying microsatellites, as it allows for specifying the minimum and maximum motif sizes in the parameters.

Etandem/Equicktandem programs (Rice et al., 2000) are components of the EMBOSS package, developed by Richard Durbin, utilized in tandem repeat identification in DNA. Equicktandem identifies repeats for each pattern size up to a specified limit, whereas Etandem, which should be employed after the other tool, computes a potential consensus of the repeated pattern. The tools support a wide range of output formats, and they apply to SSRs, as they allow for the definition of maximum and minimum motif sizes and enable the study of perfect and/or imperfect SSRs by including a parameter for mismatches.

Mreps (Kolpakov et al., 2003) employs a mixed combinatorial/heuristic approach to identify repeats of all possible sizes within a single program run. The output includes detailed information about each repeat, such as start and end positions, size, period, exponent, error level, and the repeat sequence. The web interface provides visualizations of repeat alignments. For SSR analyses, mreps allows users to define motif size and iteration parameters.

STAR (Delgrange and Rivals, 2004) is capable of identifying significant approximate (imperfect) tandem repeats of a given motif in DNA sequences. It distinguishes between exact tandem repeats (ETRs), which result from the tandem duplication of the motif, and approximate tandem repeats (ATRs), which arise from ETRs through point mutations. While STAR can be utilized to investigate microsatellites, its primary objective is to identify specific motifs pre-defined by the user within sequences, rather than to identify all SSRs.

T-reks (Jorda and Kajava, 2009) is designed primarily for the analysis of TR in proteins, although it also works with DNA sequences. It offers a standalone mode with a user-friendly graphical interface for local use and a web interface version. However, the web interface is limited to approximately 100,000 amino acids as input, does not allow file upload, or the definition of parameters such as minimum and maximum repeat sizes. This may be an issue for studies that focus on SSRs.

Tandem Repeats Finder (TRF) (Benson, 1999) is a software program that can be used to locate and display TR in DNA sequences. TRF allows users to restrict their search to small motifs of 1–5 period sizes, which is useful for SSR research. However, TRF does not offer an option to search for perfect arrays only, as it considers mismatches in its default parameters. One advantage of TRF is that users are not required to specify the pattern, pattern size, or any other parameter.

#### 3.2.2 Tools specific for detecting microsatellites

A total of 33 tools specifically designed for SSR identification were identified, including 11 basic tools and 22 pipeline tools.

##### 3.2.2.1 Basic tools

###### 3.2.2.1.1 Basic tools for p-SSR

A comparison of the seven tools that focus on perfect SSRs (Table 4) revealed that none of them have the integrated primer design function. All of them can be executed via command line on Linux; however, only GMATo (Wang et al., 2013) and PERF (Avvaru et al., 2018) can also be executed on Windows and MacOS, with GMATo being the only one with a graphical interface for local executions. Additionally, only SSRIT (Temnykh et al., 2001) and Misa (Thiel et al., 2003) have web versions. Despite focusing on p-SSRs, GMATo and Kmer-SSR (Pickett et al., 2017) do not define a limit for the maximum motif size identified. Others, such as Misa, SA-SSR (Pickett et al., 2016), and SSRIT (Temnykh et al., 2001), allow the analysis of motifs larger than 6 bp. Among these, only PERF (Avvaru et al., 2018) can use FastQ files as input and generate user-friendly chart outputs.

**TABLE 4:** Tools for detecting SSR part 1 – basic tools for perfect SSR analysis.

Name	Reference/Year	Mining tool	Integrated toolset	Platform	Execution	Focus	Max motif lenght	Input files	Output graphs/Charts	p-SSR	i-SSR	c-SSR	Primer design	Tool’s link
GMATo	Wang 2013	GMATo	No	Linux, Mac OS, Windows	Graphical Interface and Command Line	SSR	No limit	FASTA	No	Yes	No	No	No	https://sourceforge.net/projects/gmato/files/?source=navbar
Kmer-SSR	Pickett 2017	K-mer-SSR	No	Linux	Command Line	SSR	No limit	FASTA	No	Yes	No	No	No	https://github.com/ridgelab/Kmer-SSR
Misa	Thiel 2003	MISA	No	Linux, Web	Graphical Interface and Command Line	SSR	1–6 bp	FASTA	No	Yes	No	Yes	No	https://webblast.ipk-gatersleben.de/misa/
PERF	Avvaru 2018	PerfSSR	No	Linux, Windows, Mac OS	Command Line	SSR	1–6 bp	FASTA; FASTQ	Yes	Yes	No	No	No	https://github.com/rkmlab/perf
SA-SSR	Pickett 2016	SA-SSR	No	Linux	Command Line	SSR	1–7 bp	FASTA	No	Yes	No	No	No	https://github.com/ridgelab/SA-SSR
SSRIT (CugiSSR)	Temnykh 2001	SSRIT	No	Linux, Web	Graphical Interface and Command Line	SSR	2–10 bp	DNA Sequence	No	Yes	No	No	No	https://archive.gramene.org/db/markers/ssrtool
TROLL	Castelo 2002	Troll	No	Linux	Command Line	SSR	1–6 bp	DNA sequence	No	Yes	No	No	No	https://sourceforge.net/projects/finder/

“Yes” has been highlighted in the columns for visualization purposes. “SSR” = Simple Sequence Repeats or microsatellite. “BP” = Base pair. “p-SSR” = Perfect SSR. “i-SSR” = Imperfect SSR. “c-SSR” = Compound SSR.

GMATo (Wang et al., 2013) stands out by providing graphical interfaces for SSR mining through local execution. Also, c-SSR and i-SSR can be derived from the SSR loci output using additional scripts. GMATo outputs p-SSR reports and statistical distribution files in tab-delimited plain text format for easy import into other applications. An interesting point to note is that the developers of GMATo identified the need for future development to include additional functions. To address this, they launched a new pipeline tool named GMATa (Wang and Wang, 2016), with a novel algorithm, integrating features such as graphical display of statistical data and primer designing.

Kmer-SSR (Pickett et al., 2017) and SA-SSR (Pickett et al., 2016) are tools developed by the same author that share similar execution, parameters, and output format. However, they utilize different algorithms for SSR discovery in large genetic sequences. The SA-SSR employs a suffix array-based algorithm, while the Kmer-SSR’s algorithm is based on k-mer decomposition. In its tool validation section, the Kmer-SSR demonstrated better benchmark test performance than the SA-SSR. Moreover, Kmer-SSR offers a range of filters for analysis, including those based on atomicity, cyclic duplicates, enclosed SSRs, minimum SSR length, and specific SSR period sizes.

Misa (Thiel et al., 2003) identifies p-SSRs and c-SSRs in fasta sequences, providing the SSRs identified and statistics. Despite not having an integrated primer design feature, MISA provides supplementary scripts for command line execution to process outputs in Primer3 (Untergasser et al., 2012). The web version (Beier et al., 2017) limits input files to 2 Mb and does not provide online result visualization, emailing the results instead. Misa does not support batch processing, requiring users to handle files individually or as a multifasta file. Advanced users often create custom batch scripts to address this limitation.

PERF (Avvaru et al., 2018) compares direct strings to repeat to avoid missing overlapping or mid-motif SSRs in Fasta or FastQ sequences. It accepts both GFF and GTF format files and can classify SSRs as Genic, Exonic, Intronic, or Intergenic based on their position. Despite being a command-line tool, PERF provides user-friendly, post-processed outputs. It can produce interactive HTML reports, including a bar chart displaying the 10 most frequent repeats, a depiction of the relative distribution of repeats categorized by motif length, and a line chart illustrating the relationship between length and frequency of selected repeats.

SSRIT (Temnykh et al., 2001) provides a command-line and web-based tool. However, the command-line version was not accessible via the provided link on their website. The webtool, though user-friendly, presented limitations. It only accepts pasted sequences in fasta format and allows the selection of the maximum motif length (2–10 nucleotides) and minimum number of repeats as parameters, without the option to set different minimum repeats for each motif length. The results are displayed in tabular format, showing the motif, number of repeats, SSR start, and SSR end. Despite these constraints, the platform is efficient and straightforward, suitable for rapid online analyses.

TROLL (Castelo et al., 2002), despite its name, is primarily focused on identifying p-SSRs using a pre-defined dictionary containing all possible SSR motifs. Its execution requires input arguments specifying the minimum length of SSRs, the maximum motif length, and files containing the motif list and DNA sequence. The output includes the starting position of each repeat, the motif, and the repeat length in base pairs (bp). The resulting file can be readily imported into a variety of applications and spreadsheets. Additionally, customized filter scripts can be created to process TROLL’s output.

###### 3.2.2.1.2 Basic tools for p-SSR and i-SSR analysis

Among the four basic tools capable of identifying i-SSRs, summarized in Table 5, none integrated primer design. Although IMEx (Mudunuri and Nagarajaram, 2007; Mudunuri et al., 2010a) claims to have this functionality in its web version, this version is currently non-functional. However, IMEx (Mudunuri et al., 2010a) provides a graphical interface for local executions and identifies SSRs with motifs ranging from 1 to 6 bp. In contrast, Imperfect SSR Finder (Stieneke and Eujayl, 2007) and Microsatellite Repeats Finder (Bikandi, 2006) are web tools that do not detect mononucleotides, but can analyze motifs up to 10 bp. None of the tools accept FastQ input files. Only IMEx can determine if the SSR is in a coding or non-coding position by accepting PTT files. Furthermore, the only two tools that can identify c-SSRs are IMEx and Imperfect SSR Finder. Additionally, none of the tools generate graphs or charts.

**TABLE 5:** Tools for detecting SSR part 2 – basic tools for perfect and Imperfect SSR analysis.

Name	Reference/Year	Mining tool	Integrated toolset	Platform	Execution	Focus	Max motif lenght	Input files	Output graphs/Charts	p-SSR	i-SSR	c-SSR	Primer design	Tool’s link
IMEx	Mudunuri 2007	IMEx	No	Linux, Web	Graphical Interface and Command Line	SSR	1–6 bp	FASTA; PTT	No	Yes	Yes	Yes	No	http://www.mcr.org.in/IMEX/download.html
Imperfect SSR Finder	Stieneke 2007	Imperfect SSR Finder	No	Web	Graphical interface	SSR	2–10 bp	FASTA	No	Yes	Yes	Yes	No	https://ssr.nwisrl.ars.usda.gov/
Microsatellite repeats finder	Bikandi 2006	Microsatellite repeats finder	No	Web	Graphical Interface	SSR	2–10 bp	DNA Sequence	No	Yes	Yes	No	No	http://insilico.ehu.es/mini_tools/microsatellites/?info
Sputnik	Morgante 2002	Sputnik	No	Linux, Windows	Command Line	SSR	1–5 bp	FASTA	No	Yes	Yes	No	No	http://wheat.pw.usda.gov/ITMI/EST-SSR/LaRota/

“Yes” has been highlighted in the columns for visualization purposes. “SSR” = Simple Sequence Repeats or microsatellite. “BP” = Base pair. “p-SSR” = Perfect SSR. “i-SSR” = Imperfect SSR. “c-SSR” = Compound SSR.

IMEx (Mudunuri and Nagarajaram, 2007) is an effective tool for identifying p-SSR, i-SSR, and c-SSR. Its graphical interface for local execution, inclusion of flanking regions in the output for primer design, support for batch mode input files, and generation of HTML and text outputs distinguish it from other similar software. IMEx aligns each repeat with its consensus sequence and can classify SSRs as coding or non-coding. The primary limitations include the numerous customizable parameters and the requirement for a PTT format file for analyzing coding regions, which might pose challenges for those accustomed to GenBank format annotations. However, these limitations are addressed by the user-friendly pipeline web tool EasySSR (Alves et al., 2023), which runs IMEx as a mining algorithm.

Imperfect SSR Finder (Stieneke and Eujayl, 2007) is a webtool based on the SSRIT algorithm, that has been modified to identify p-SSR, i-SSR, and c-SSR. Despite its user-friendly interface and capacity to seemingly handle inputs of any size, it lacks mononucleotide repeat detection and may initially seem complex due to its extensive parameter range. For i-SSR and c-SSR, the user can define a non-repeating region (NRR), which is the maximum distance of seemingly random nucleotides separating the SSRs. In this context, NRR might be considered synonymous with mismatches or with dMAX. One drawback is that it does not include flanking regions in its output.

Sputnik repeat-finder tool, developed by Chris Abajian in 1994 (unpublished data), is designed to identify microsatellites with a customizable deviation from a perfect repeat. Two modified versions of the tool have been developed: Modified Sputnik I (Morgante et al., 2002) and Modified Sputnik II (La Rota et al., 2005). The most recent version identified was that of La Rota, but some pipelines tools like PolySSR (Tang et al., 2008) and SSRpoly (Duran et al., 2013) also employ Sputnik as a basic tool for SSR mining with potential additional modifications. Modified Sputnik II has versatile parameters, adjusts FASTA sequence header parsing, and formats reports for direct database import. Its command-line interface, however, may pose challenges for inexperienced users.

Microsatellite repeats finder (Bikandi, 2006) is a web-based tool designed for quick analysis of perfect p-SSR and i-SSR. While the source code is available for users, the platform primarily operates online, limiting sequence analysis to 100,000 bp. The tool is straightforward, with few parameters. The output is also simple, displaying the SSR start position, their class, the number of iterations, and the repeated sequence. This tool may be of benefit to researchers seeking a rapid assessment of SSR presence within their sequences.

##### 3.2.2.2 SSR pipeline tools

This study identified 22 functional pipeline tools for SSR analysis, with 18 of these incorporating primer design functionality. Among the 22 tools identified, 14 are exclusively focused on the analysis of p-SSRs, while the remaining 8 are designed to analyze both perfect and imperfect SSRs.

###### 3.2.2.2.1 Pipeline tools for p-SSR analysis

Regarding the 14 pipeline tools with focus on the identification of perfect SSRs, as summarized in Table 6, it was observed that all the pipeline tools integrate an SSR mining algorithm with other functions. It should be noted that most integrate Primer3 (Untergasser et al., 2012) and have the native primer design function, except MICAS (Sreenu et al., 2003) and SSRenricher (Luo et al., 2020). Six tools employ original algorithms for SSR mining, while eight utilize consolidated basic tools as mining algorithms. Among these, MICAS (Sreenu et al., 2003) and WebSat (Martins et al., 2009) are the only ones that do not integrate Misa (Thiel et al., 2003), employing instead IMEx (Mudunuri and Nagarajaram, 2007) and Troll (Castelo et al., 2002), respectively, as their basic tools.

**TABLE 6:** Tools for detecting SSR part 3 – pipeline tools for perfect SSR analysis.

Name	Reference/Year	Mining tool	Integrated toolset	Platform	Execution	Focus	Max motif lenght	Input files	Output graphs/Charts	p-SSR	i-SSR	c-SSR	Primer design	Tool’s link
CandiSSR	Xia 2016	MISA	BLAST, Primer3 and Clustalw	Linux	Command Line	SSR	2–6 bp	FASTA	No	Yes	No	Yes	Yes	https://github.com/xiaenhua/CandiSSR
FullSSR	Metz 2016	FullSSR	Primer3	Linux	Command Line	SSR	1–5 bp	FASTA	Yes	Yes	No	No	Yes	https://sourceforge.net/projects/fullssr/
GMATa	Wang 2016	GMATa	Primer3, e-PCR	Linux, Mac OS, Windows	Graphical Interface and Command Line	SSR	No limit	FASTA; FASTQ	Yes	Yes	No	No	Yes	https://sourceforge.net/projects/gmata/?source=navbar
MegaSSR	Mokhtar 2023	MISA	Primer3, Primersearch	Linux, Web	Graphical Interface and Command Line	SSR	1–6 bp	FASTA; GFF	Yes	Yes	No	Yes	Yes	https://bioinformatics.um6p.ma/MegaSSR
MICAS	Sreenu 2003	IMEx	IMEx, MICdb3.0	Web	Graphical Interface	SSR	1–10 bp	FASTA; PTT	No	Yes	No	No	No	http://www.mcr.org.in/micas/
Micro-Primers	Alves 2022	MISA	Trimmomatic, Cutadapt, FLASH, CD-HIT, Primer3	Linux, Mac OS	Graphical Interface and Command Line	SSR	No limit	FASTQ	No	Yes	No	Yes	Yes	https://github.com/FilAlves/micro-primers
MiMi	Fox 2019	PALfinder	Muscle, Primer3	Linux, Mac OS	Command Line	SSR	1–6 bp	FASTA; FASTQ	No	Yes	No	No	Yes	https://github.com/graemefox/mimi
msatcommander	Faircloth 2008	msatcommander	Primer3	Linux, Mac OS, Windows	Graphical Interface and Command Line	SSR	1–6 bp	FASTA	No	Yes	No	No	Yes	https://code.google.com/archive/p/msatcommander/
PALfinder	Castoe 2012	PALfinder	Primer3, RepBase	Linux, Mac OS	Command Line	SSR	2–6 bp	FASTA; FASTQ	No	Yes	No	No	Yes	https://sourceforge.net/projects/palfinder/
PolyMorphPredict	Das 2019	MISA	Primer3, e-PCR	Web	Graphical Interface	SSR	1–6 bp	FASTA	No	Yes	No	Yes	Yes	http://webtom.cabgrid.res.in/polypred/
SSR2Marker	Yue 2022	MISA	BLAST, MAFFT, Primer3 and e-PCR	Linux	Command Line	SSR	1–6 bp	FASTA; MISA	No	Yes	No	Yes	Yes	https://github.com/aaranyue/SSR2Marker
SSRenricher	Luo 2020	MISA	MISA, CD-HIT, Muscle	Linux, Mac OS	Graphical Interface and Command Line	SSR	1–6 bp	FASTQ	No	Yes	No	Yes	No	https://github.com/byemaxx/SSREnricher
SSRMMD	Gou 2020	SSRMMD	Primer3	Linux, Mac OS, Windows	Command Line	SSR	1–6 bp	FASTA	No	Yes	No	Yes	Yes	https://github.com/GouXiangJian/SSRMMD
Websat (WebTROLL)	Martins 2009	Troll	Troll, Primer3	Web	Graphical Interface	SSR	1–6 bp	FASTA	No	Yes	No	No	Yes	https://bioinfo.inf.ufg.br/websat/

“Yes” has been highlighted in the columns for visualization purposes. “SSR” = Simple Sequence Repeats or microsatellite. “BP” = Base pair. “p-SSR” = Perfect SSR. “i-SSR” = Imperfect SSR. “c-SSR” = Compound SSR.

In terms of execution, as detailed in table 6, there is a diverse range of profiles among the tools. Some, like WebSat (Martins et al., 2009), are solely web-based, while others, such as SSRMMD (Gou et al., 2020) and MiMi (Fox et al., 2019), are exclusively for local execution via command lines. Additionally, tools like Micro-primers and GMATa (Wang and Wang, 2016) offer graphical user interfaces for local executions, and tools like Misa (Thiel et al., 2003) and MegaSSR (Mokhtar et al., 2023) provide web servers and execution via command line.

Most tools target SSRs ranging from 1 to 6 base pairs. However, notable exceptions include GMATa (Wang and Wang, 2016) and Micro-primers (Alves et al., 2022), which do not impose size restrictions on the repeats, and PALfinder (Castoe et al., 2012) and CandiSSR (Xia et al., 2016), which specifically exclude mononucleotide repeats. Moreover, an increase in the number of analytical tools capable of processing FastQ files, identifying c-SSRs, and generating graphical representations such as charts and graphs as part of their output was observed. Following the structure used so far, some unique features of each tool will be highlighted.

CandiSSR (Xia et al., 2016) is a pipeline that integrates Misa (Thiel et al., 2003) with BLAST (Altschul et al., 1997), Primer3 (Untergasser et al., 2012), and Clustalw (Thompson et al., 2003). Its purpose is to automatically identify candidate polymorphic SSRs (polySSRs) from multiple assembled sequences. It can identify putative polySSRs from transcriptome datasets and assembled genome sequences. The pipeline provides two confidence metrics (standard deviation and missing rate of SSR repetitions) to assess polySSR feasibility. It automatically designs primer pairs and evaluates primer-binding region similarities for successful marker development. The output includes detailed information on candidate PolySSRs, primer pairs, and flanking sequences for further genetic studies.

FullSSR (Metz et al., 2016) is a pipeline designed to identify microsatellite loci by using their algorithm and design primers with primer3 (Untergasser et al., 2012). The tool produces three types of output: a clear HTML report, Primer3 native outputs, and individual text files for each identified SSR and their respective primers. The publication that released the FullSSR tool highlights that in comparison to MISA (Thiel et al., 2003), FullSSR has superior capability in identifying SSRs suitable for primer design, as many SSRs identified by Misa may be unsuitable for primer design due to their proximity to sequence ends, precluding primer design for both sides.

GMATa (Wang and Wang, 2016) is a pipeline tool developed by the same authors as GMATo (Wang et al., 2013), but with its own novel SSR mining algorithm and integrating Primer3 (Untergasser et al., 2012), and e-PCR (Schuler, 1997). It accepts Fasta and FastQ files, with a workflow of six modules that can be seamlessly integrated and executed automatically or independently. GMATa provides graphical display within a genome browser and produces various user-friendly outputs, such as SSR loci results, SSR statistical analysis and graphic plotting, genome wide SSR marker design, marker mapping results, and polymorphisms these outputs facilitate the generation of high-quality statistical data that is suitable for direct integration into publications.

MegaSSR (Mokhtar et al., 2023) is a novel tool that is available as a web server and a standalone pipeline. It utilizes Misa (Thiel et al., 2003) as the basic tool and integrates the results with Primer3 (Untergasser et al., 2012) and Primersearch (Rice et al., 2000), which enables the design of PCR-based primers and *in silico* PCR validation. The tool accepts as input fasta files, and additional annotations in gff or gff3 format if the user wants to locate coding or non-coding regions. The results of MegaSSR can be accessed through an interactive HTML page that includes graphs and tables containing various aspects of SSR markers and corresponding PCR primers. A limitation of the tool, however, is its single-file analysis capability.

MICAS (Sreenu et al., 2003) is a web server designed for the study of non-redundant microsatellites in bacterial or archaeal genomes. Their integrated webtool for SSR mining is no longer fully functional, preventing users from identifying new SSRs in MICAS. Nevertheless, MICAS 3.0 also integrates MICdb3.0 (Mudunuri et al., 2014), which contains SSR information from over 5,000 prokaryotic genome sequences. This functionality enables users to explore and compare SSR distributions and perform pairwise genome comparisons. Users can export data in various formats for further analysis. Despite the limitations in new SSR identification, MICAS remains a valuable resource for studying microsatellites in prokaryotic genomes, providing access to a vast database of pre-extracted data.

Micro-primers (Alves et al., 2022) integrates Misa with Trimmomatic (Bolger et al., 2014), Cutadapt (Martin, 2011), FLASH (Magoč and Salzberg, 2011), CD-HIT (Li and Godzik, 2006), and Primer3 (Untergasser et al., 2012) to identify and design PCR primers for amplifying SSR loci. It takes as input a FASTQ file containing sequences (reads) from NGS (next-generation sequencing). The output is a text file with information about the microsatellite markers, including the number of alleles, melting temperature, and corresponding primer set products, which facilitates marker selection. It can be executed via command line or a graphical interface. Therefore, it could be a valuable tool for researchers analyzing newly sequenced data, for metagenomic projects, or integration into other pipelines.

Msatcommander (Faircloth, 2007) is a tool designed to facilitate the identification of SSR by integrating their original algorithm with primer3 (Untergasser et al., 2012) for primer design and primer tagging through an automated process. It offers a local graphical user interface or command line for execution. The results and primers are outputted in CSV format for easy integration with spreadsheet or database programs. Primer-specific files generated by Primer3 are provided in TXT format, compatible with spreadsheet, database, or text-editor applications.

PALfinder (Castoe et al., 2012) is a command-line pipeline for the automated analysis of assembled genomes and sequencing reads, to identify SSRs and design PCR primers for potentially amplifiable SSR loci (PALs). It integrates an original SSR mining algorithm with Primer3 (Untergasser et al., 2012) and RepBase (Jurka et al., 2005), accepting Fasta and FastQ, formats for input, including Illumina paired-end reads and 454 single-end reads. PALfinder identifies SSR-containing reads and selects flanking sequences suitable for PCR primer sites, resulting in PALs presented in a tab-delimited format with primer information. Controlled through a customizable parameter settings file, PALfinder is valuable for research groups analyzing SSRs directly from sequencing reads and can be integrated into other pipelines, as demonstrated by its integration into the MiMi pipeline (Fox et al., 2019).

MiMi (Fox et al., 2019) enhances SSR mining efficiency by incorporating the PALfinder (Castoe et al., 2012) pipeline and the Muscle alignment tool (Edgar, 2004). It can use Fasta and FastQ files as input and aims to compare genomic data from multiple individuals of the same species, a departure from using data from a single individual. The software allows for the *in silico* identification of polymorphic loci and other key characteristics of potential microsatellite markers. It also enables marker amplification by PCR and reduces the number of markers requiring laboratory testing for polymorphism, thereby improving overall marker development success. Furthermore, MiMi enables clear visualization and avoidance of insertion/deletions in flanking regions when designing microsatellite panels, which is a significant source of error.

PolyMorphPredict (Das et al., 2019) integrates Misa (Thiel et al., 2003) with Primer3 (Untergasser et al., 2012) and e-PCR (Schuler, 1997) to mine microsatellite loci and compute primers from genome/transcriptome data of any species, focusing however in agriculture research. It performs e-PCR using published primers for polymorphism discovery and across species transferability of microsatellite loci. The tool also compares various whole genome sequences and their genotypes to discover microsatellite loci, identify polymorphic loci, and design primers for rapid genotyping. The tool is only available as a web service, which occasionally malfunctions. However, it is a valuable tool for designing primers for polymorphic SSR markers.

SSR2marker (Yue and Liu, 2022) is a new pipeline that integrates Misa (Thiel et al., 2003) with BLAST (Altschul et al., 1997), MAFFT (Nakamura et al., 2018), Primer3 (Untergasser et al., 2012), and e-PCR (Schuler, 1997) to explore polymorphic SSR markers between any two given sequences. It identifies monomorphic and dimorphic SSR markers, providing detailed information for genetic analyses and marker-assisted breeding, such as SSR motifs, primer pairs, amplified fragments, sequence sizes, length polymorphisms, and statistical calculations, to facilitate subsequent genetic analyses and marker-assisted breeding for example,. SSR2marker is a versatile tool that can significantly reduce processing time for scientists aiming to identify markers and develop primers from sequence comparisons.

SSRenricher (Luo et al., 2020) is optimized for polymorphic SSR enrichment in transcripts, rather than primer design and has a graphical user interface. The integration of Misa (Thiel et al., 2003) and CD-HIT (Li and Godzik, 2006) with additional scripts enables the execution of six core analysis steps: SSR mining, sequence clustering, sequence modification, enrichment containing polymorphic SSR sequences, false-positive removal, and results output and multiple sequence alignment. Collectively, these steps facilitate the identification and analysis of polymorphic SSRs in transcripts. This tool may be particularly pertinent for groups engaged in transcriptomics studies.

SSRMMD (Gou et al., 2020) is a command-line pipeline with a proprietary algorithm for mining p-SSR loci and potential polymorphic SSRs from assembled sequences like genomes or transcriptomes. It facilitates primer design by providing a native tool called connectorToPrimer3 to link SSRMMD with Primer3 (Untergasser et al., 2012). To identify candidate polymorphic SSRs, at least two assembled sequences are required. The SSRMMD algorithm assesses the conservatism and uniqueness of SSR flanking sequences for the identification of polymorphic SSRs, generating comprehensive information records and statistical analyses of SSRs, along with records of candidate polymorphic SSRs.

Websat (Webtroll) (Martins et al., 2009) is a user-friendly webtool that integrates TROLL (Castelo et al., 2002) for SSR mining and Primer3 (Untergasser et al., 2012) for primer design. Users submit sequences in raw or FASTA format (max upload 150,000 characters). The output highlights SSRs in yellow and underlines them in a table with numbered lines and spaces to aid SSR coordinate location. Clicking on an SSR invokes primer design to generate flanking primers. Users can modify primer3 parameters for custom design. Successful primers are green, SSRs blue, and failed designs are reported. All primers can be downloaded in a. csv file with SSR info, product size, sequences, and melting temperatures.

###### 3.2.2.2.2 Pipeline tools for p-SSR and i-SSR analysis

This topic will analyze eight pipeline tools that, in addition to mining p-SSRs, also provide options to identify i-SSRs. Table 7 indicates that five pipeline tools utilize consolidated basic tools as SSR mining algorithms, while the remaining three use proprietary algorithms. It was further observed that two tools employ Sputnik (La Rota et al., 2005), while the others each use a different algorithm for SSR mining. All tools, except for EasySSR (Alves et al., 2023) and SSR_pipeline (Miller et al., 2013), incorporated integrated primer design functionality.

**TABLE 7:** Tools for detecting SSR part 4 – pipeline Tools for perfect and imperfect SSR analysis.

Name	Reference/Year	Mining tool	Integrated toolset	Platform	Execution	Focus	Max motif lenght	Input files	Output graphs/Charts	p-SSR	i-SSR	c-SSR	Primer design	Tool’s link
BatchPrimer3	You 2008	SSR search	Primer3	Linux, Mac OS, Windows, Web	Graphical Interface and Command Line	SSR	2–6 bp	FASTA	No	Yes	Yes	No	Yes	http://wheat.pw.usda.gov/demos/BatchPrimer3/
EasySSR	Alves 2023	IMEx	EasySSR	Linux, Web	Graphical Interface	SSR	1–6 bp	FASTA; GBK	Yes	Yes	Yes	Yes	No	https://computationalbiology.ufpa.br/easyssr/
IDSSR	Guang 2019	SSRIT	SOAPindel, BLAST, Primer3	Linux	Command Line	SSR	2–6 bp	FASTA; FASTQ	Yes	Yes	Yes	No	Yes	https://github.com/Allsummerking/IDSSR
Krait	Du 2018	Krait	Primer3	Linux, Mac OS, Windows	Graphical Interface	SSR	1–6 bp	FASTA; GTF; GFF	No	Yes	Yes	Yes	Yes	https://github.com/lmdu/krait
PolySSR	Tang 2008	Sputnik	Primer3, Cross_match, CAP3, CheckSSR	Linux	Command Line	SSR	2–6 bp	FASTA	No	Yes	Yes	No	Yes	http://www.bioinformatics.nl/tools/polyssr/
SciRoKo	Kofler 2007	SciRoKo	Primer3	Linux, Mac OS, Windows	Graphical Interface and Command Line	SSR	2–6 bp	FASTA	No	Yes	Yes	Yes	Yes	https://kofler.or.at/bioinformatics/SciRoKo/index.html
SSR_pipeline	Miller 2013	SSR_pipeline	SSR_pipeline	Linux, Mac OS, Windows	Command Line	SSR	1–6 bp	FASTA; FASTQ	No	Yes	Yes	Yes	No	https://pubs.usgs.gov/ds/778/
SSRpoly	Duran 2013	Sputnik	Sputnik, MySQL, SSRPrimer	Linux	Command Line	SSR	2–6 bp	FASTA	No	Yes	Yes	No	Yes	https://appliedbioinformatics.com.au/Edwards/index.php/SSRPoly

“Yes” has been highlighted in the columns for visualization purposes. “SSR” = Simple Sequence Repeats or microsatellite. “BP” = Base pair. “p-SSR” = Perfect SSR. “i-SSR” = Imperfect SSR. “c-SSR” = Compound SSR.

Only SciRoKo (Kofler et al., 2007), Krait (Du et al., 2018), EasySSR (Alves et al., 2023), and BatchPrimer3 (You et al., 2008) were executable through a graphical interface, with the latter two being the sole tools to offer web servers. Most tools focused on SSRs with 1-6 base pairs, although some excluded mononucleotides. Regarding file formats, only IDSSR (Guang et al., 2019) and SSR_pipeline (Miller et al., 2013) accepted FastQ files as input, while only Krait (Du et al., 2018) and EasySSR (Alves et al., 2023) allowed the use of annotation files to analyze SSRs in the coding/non-coding context. Outputs containing graphics and charts were provided exclusively by EasySSR (Alves et al., 2023) and IDSSR (Guang et al., 2019).

BatchPrimer3 (You et al., 2008) while primarily designed for primer design, was also considered as an SSR pipeline tool. It can identify SSRs within a genome provided in FASTA format by integrating with Primer3 (Untergasser et al., 2012) with SSR search (Nicot et al., 2004) as the basic tool to identify SSRs This basic tool, however, was not detailed in this paper due to the current unavailability of its source code (Table 2). SSR primers are selected from flanking regions, with flexible screening criteria typically detecting motifs from dinucleotide to hexanucleotide repeats. Output includes a main HTML page summarizing primer design, an HTML table of designed primers, a tab-delimited text file with the same information, and a detailed primer view page for each sequence.

EasySSR (Alves et al., 2023) is a user-friendly web server for large-scale batch analyses, enabling the comparison of genomic data from multiple individuals in a single run. It accepts one or more fasta files as input, with optional GBK files for coding region identification, which are converted to PTT files for output. EasySSR executes IMEx in batches (Mudunuri and Nagarajaram, 2007) for p-SSR identification in every file, and optionally detects i-SSR and c-SSRs. The IMEx raw results are made available to users and are automatically processed and compared via additional scripts. In the HTML page displaying the results, EasySSR offers customizable flanking regions for use in primer design and provides additional processed outputs such as interactive tables and graphs, which show statistical and comparative results among genomes, along with suggested SSR markers.

IDSSR (Guang et al., 2019) is a tool with focus at identifying polymorphic SSRs by integrating SSRs with nucleotide insertions/deletions (INDEL) solely based on a single genome sequence and paired-end reads. Input files include assembled genome sequences in FASTA format and sequenced clean reads in FASTQ format. The tool employs SSRIT (Temnykh et al., 2001) as its SSR mining tool, which is focused on identifying perfect SSRs. However, the tool also integrates SOAPindel (Li et al., 2013), BLAST (Altschul et al., 1997), and Primer3 (Untergasser et al., 2012), which enables it to identify i-SSR as well. A limitation is that INDEL markers require short insert sizes and 25× genome coverage, potentially limiting IDSSR’s use. Nonetheless, IDSSR provides valuable data for further analysis, including repeat motifs, positions, chromosome locations, annealing temperatures, and primer sequences.

Krait (Du et al., 2018) is a user-friendly tool that can be executed directly from a desktop application with a graphical interface. It can identify p-SSR, i-SSR and c-SSR in whole genomic sequences, and design primers by integrating their proprietary algorithm with Primer3 (Untergasser et al., 2012). Krait can also locate SSRs within gene coding regions if GTF or GFF annotation files are provided as input. Additionally, Krait provides statistical data for interpretation and the outputs can be exported in formats such as Fasta, GFF3, or CSV. The tables provided by the tool are easy to navigate and include filters, making it an intuitive tool.

PolySSR (Tang et al., 2008) and SSRpoly (Duran et al., 2013), despite their similar names and attributes, are distinct tools with no shared publication teams. Both tools have Sputnik (La Rota et al., 2005) as their SSR mining tool, focusing on identifying and designing primers for polymorphic SSRs in EST sequences using cluster-based strategies. PolySSR (Tang et al., 2008) offers an online database for examining polymorphic SSRs from preprocessed EST data and integrates cross_match (Ewing and Green, 1998), CAP3 (Huang and Madan, 1999), Primer3 (Untergasser et al., 2012) and CheckSSR (Tang et al., 2008), to conduct sequence alignment, vector removal with EST, sequence clustering, polymorphic SSR prediction, and primer design. However, accessing PolySSR is limited, requiring users to request access to the download link via email. In contrast, SSRpoly (Duran et al., 2013) is easily downloadable from its website, functioning similarly to PolySSR (Tang et al., 2008), by using a custom method for polymorphic SSR prediction and SSRPrimer (Robinson et al., 2004) for primer design.

SciRoKo (Kofler et al., 2007) is a tool for identifying perfect, imperfect, and compound SSRs. It offers a standalone user-friendly tool with a graphical interface, including an SSR search module with five search modes and an SSR statistics module with three classification and statistics options. Although SciRoKo does not directly integrate Primer3, it provides a DesignPrimers module for automated PCR primer design using Primer3 (Untergasser et al., 2012) and SciRoKo. Additionally, SciRoKoCo is the command line version for the SciRoKo SSR-search module.

SSR_pipeline (Miller et al., 2013) is a command-line tool that uses original algorithm for identifying SSRs from Illumina DNA sequencing data and other platforms like Roche 454 and Ion Torrent. The program was designed for analyses of paired-end sequences from low-coverage whole genomic DNA libraries. It comprises three analysis modules and a control module for automating large data analyses. These modules are used to identify high-quality paired-end sequences, align paired-end reads into composite DNA sequences, and identify microsatellite-containing sequences based on user-defined parameters. While the pipeline does not include a primer design function, it provides flanking regions in its output for primer design in other tools.

## 4 Discussion

### 4.1 Why are there so many different tools?

The proliferation and diversity of tools aimed at discovering simple sequence repeats (SSRs) are intricately linked to the complex nature of genomic research (Lerat, 2010). Various factors contribute to this diversity, with each tool designed to address specific limitations of existing tools (Avvaru et al., 2018). Here, will be discussed key aspects to consider when selecting an SSR mining tool. All data is available in Supplementary Table S1.

#### 4.1.1 Availability

When users search for a tool, they expect it to be functional and accessible. However, some tools are no longer available due to various reasons, such as discontinued web servers, broken links, or lack of maintenance (Alves et al., 2023). Among the 74 tools identified in this study, 43% were no longer functional. Therefore, the remaining 42 active tools were filtered and analyzed to help users.

#### 4.1.2 Basic tools and pipelines

Another important aspect analyzed for the variety of tools is the existence of basic tools and pipeline tools. Although they perform the function of SSR identification, many variations of algorithms are made to obtain more accurate results in less time (Pickett et al., 2017). Furthermore, many authors feel the need to go beyond SSR identification (Bizzaro and Marx, 2003), creating new basic tools and pipelines with new features such as batch analysis, support for a variety of inputs, graphical outputs, statistics, primer design, and polymorphism studies (Mudunuri et al., 2010b). This study identified 38 pipeline tools and 36 basic tools, reflecting the diverse range of functionalities available in SSR research tools.

#### 4.1.3 Execution

The method of tool execution is a crucial factor for users. Some prefer command line interfaces for speed and integration capabilities, especially for batch analyses and pipeline integration (Yue and Liu, 2022). Others find graphical interfaces more intuitive and user-friendly (Alves et al., 2023; Mokhtar et al., 2023). Language barriers and limited command line knowledge can hinder tool use (Alves et al., 2023). Additionally, some users may face compatibility issues with their machines, leading them to prefer web-based tools (Das et al., 2019). Among the tools evaluated, 41% were command line only, 19% were graphical interface only, and 40% offered both options. Furthermore, only 43% of the available tools offered web-based analysis.

#### 4.1.4 Polymorphic SSRs and primer design

Depending on the user’s research objective, the identification of polymorphic SSRs and primer design can be crucial (Abdul-Muneer, 2014). Thus, finding tools that already have these functions integrated can save a lot of time in their research and optimize their results (Alves et al., 2022). While none of the basic tools analyzed had these functions integrated, 75% of the pipelines design primers, and some focus on polymorphic SSR, saving time and optimizing results (You et al., 2008; Xia et al., 2016). Other pipelines are focused on batch analyses, generating alignments, or providing statistics (Alves et al., 2023).

#### 4.1.5 Tool’s focus

Some tools are generalists, detecting all repetitive elements, while others specialize in tandem repeats, including not only SSRs but also, mini- and macrosatellites (You et al., 2008). Tools vary in their ability to refine analysis and may include parameters for restricting to microsatellites (Benson, 1999; Genovese et al., 2019). Users may benefit from acquiring SSR data alongside other repeats or elements, but some might prefer tools exclusively for SSRs (Sharma et al., 2007). Among the total number of tools identified, 68% were specific SSR tools, while 23% focused on Tandem Repeats and 9% on repetitive elements.

#### 4.1.6 Type of SSR

The type of SSR identified can also be an important factor in choosing the best tool because while some authors focus exclusively on the study of perfect SSRs for length polymorphism studies (Kelkar et al., 2010; Avvaru et al., 2020), others consider the inclusion of imperfect and compound SSRs to be of fundamental relevance in their results, for indel and SNP studies, for example., (Chen et al., 2011b; George et al., 2015; Ledenyova et al., 2019). Regarding SSR tools, it was observed that 64% of them focus exclusively on perfect SSRs, being unable to identify SSRs with mismatches (i-SSR or c-SSR) or excluding them from their results.

#### 4.1.7 Motif size

Most SSR tools focus on motifs with 1–6 bp, for being the widely accepted size criterion (Alves et al., 2023). However, some adopt alternative ranges, highlighting the lack of consensus (Bikandi, 2006). Some tools may extend to motifs up to 2000 bp or 5,000 bp or have no preset limits (Benson, 1999; Tarailo-Graovac and Chen, 2009). Users should choose tools based on the specific size of repetition they are analyzing.

#### 4.1.8 Algorithm and parameters

Due to algorithmic differences in the SSR mining, it is unlikely that any tools will always provide identical results (Sharma et al., 2007). The algorithm and user-defined parameters determine what the tool considers to be an SSR, leading to false positives and false negatives. Some methods cannot detect repeats that end abruptly in the middle of the motif sequence (Avvaru et al., 2018). Allowing mismatches within a tract can extend previously interrupted tracts (Behura and Severson, 2015; Alves et al., 2023). For example, the average number of perfect SSRs per genome decreased from 53.5 in an analysis that permitted only perfect SSRs to 40 in an analysis that included imperfections. These tracts were initially considered perfect but were re-labeled when mismatches were permitted (Alves et al., 2023). The parameters used by each tool were compiled in Supplementary Table S1. However, this paper does not delve into the parameters, algorithms, or benchmark testing as we believe discussions on how parameter settings of specific algorithms can impact empirical microsatellite distributions have already been made whether by the tool’s launch paper or specific review papers (Leclercq et al., 2007; Lerat, 2010). Instead, it focuses on elucidating how the programs are practically utilized, to assist users in selecting the most suitable tool for their requirements.

#### 4.1.9 Input file–type and size

Depending on the data available, researchers may find certain tools more suitable. A significant variety of input formats was observed, with FASTA being the most widely accepted format, followed by plain DNA sequences and FastQ. This highlights the availability of tools capable of identifying SSRs from sequencing reads and assembled sequences (Alves et al., 2022). Tools capable of processing specific formats like GenBank, PTT, and GFF are beneficial for researchers interested in SSRs within gene regions (Mudunuri and Nagarajaram, 2007). In addition to input type, the maximum input size accepted by a tool is crucial. For example, some web tools limit input to 150,000 nucleotides (Martins et al., 2009) or 2 MB (Beier et al., 2017),. This can be limiting for researchers with larger datasets. To overcome this, users should seek tools without input size restrictions or use command-line versions (Yue and Liu, 2022). Another factor is the number of genomes analyzed in a single run. Many tools analyze one file at a time, but researchers often need to analyze multiple genomes simultaneously. Using a multifasta file or custom scripts can address this. Researchers can also look for programs that accept multiple files as input or offer batch analysis mode (Fox et al., 2019; Alves et al., 2023).

#### 4.1.10 Output–format, content and delivery

Each researcher approaches SSR research with specific questions and objectives, seeking tools that provide efficient solutions tailored to their needs. The analysis of 74 tools revealed a diverse array of output formats, such as text, HTML, Excel tables, and graphs, each serving different user requirements (Beier et al., 2017; Alves et al., 2023). These formats enable researchers to visualize patterns and trends, integrate data, and perform gene annotation analyses. Moreover, the content of the outputs varies, including identified SSRs, polymorphic SSRs, SSR positions, statistics, abundance graphs, and primer information, allowing users to select tools that align with their data analysis and interpretation needs (Pellegrini et al., 2012). The method of delivering output is also crucial in tool selection. Some users prefer web-based tools that provide quick on-screen results, while others require data for further analysis and may prefer tools that allow for data download or access (Mokhtar et al., 2023). Additionally, considerations such as user anonymity and data delivery mechanisms, such as email, influence tool suitability. Overall, researchers should choose tools that align with their specific research questions, objectives, and preferences to ensure effective SSR analysis (Beier et al., 2017).

## 5 Conclusion

This study provides a comprehensive overview of microsatellites, aiding researchers in selecting the most appropriate tools for SSR analysis. Previously, the maximum number of tools cited in a single article was 25, while a total of 37 tools were identified from past reviews. The current study presents a comparative analysis of 74 tools for the first time, creating a significant resource for users. Additionally, a detailed supplementary table is provided, which encompasses all the data discussed in the article, as well as extra information, available for download and offline analysis with filtering capabilities. A detailed evaluation of each tool reveals the diversity of approaches and functionalities, reflecting ongoing innovation in genomic research. However, an in-depth analysis shows that each tool possesses unique characteristics, highlighting that no single tool can address all project requirements. The dynamic nature of genomics, coupled with the specific demands of various research objectives, complicates the development of a universal solution for SSR mining. Thus, this article offers multiple options for users to select tools based on their preferences, whether by literature citations or innovative functionalities. Ultimately, the selection of a tool should be guided by the study’s specific context, considering factors such as data type, research focus, and computational resources. Overall, this compilation serves as a reference for SSR tool selection and enhances the understanding of available SSR tools, contributing to more informed decision-making and promoting accurate and efficient SSR studies across diverse research areas, while underscoring the necessity for continuous refinement and innovation in this field.

## Supplementary material

SUPPLEMENTARY TABLE S1 Contains the data analyzed for this study and additional data. **(A)** contains the integrated data from the Tables 1 and 2 of the paper, with filters for easy navigation. **(B)** contains the data from Tables 1 to 8, in addition to the data from the 32 unavailable tools. **(C)** contains additional data for all 74 tools, including information on input, parameters, outputs, and limitations.
